# Factors associated with alcohol and other drug use among transgender people: a cross-sectional study, Espírito Santo, 2022-2024

**DOI:** 10.1590/S2237-96222026v35e20250205.en

**Published:** 2026-05-16

**Authors:** Juliana Almeida Moreira, Beatriz Souza Silva, Weber Eugênio Birchler, Kaio Henrique Cesconetto Coswosck, Neide Aparecida Tosato Boldrini, Valdete Regina Guandalini, José Luiz Marques-Rocha

**Affiliations:** 1 Universidade Federal do Espírito Santo, Programa de Pós Graduação em Nutrição Saúde, Vitoria, ES, Brazil Universidade Federal do Espírito Santo Programa de Pós Graduação em Nutrição Saúde Vitoria ES Brazil; 2 Universidade Federal do Espírito Santo, Centro de Ciências da Saúde, Vitória, ES, Brazil Universidade Federal do Espírito Santo Centro de Ciências da Saúde Vitória ES Brazil

**Keywords:** Gender Identity, Substance Use, Social Inequity, Gender-Affirming Surgery, Cross-Sectional Studies, Identidad de Género, Consumo de Sustancias, Inequidad Social, Cirugía de Afirmación de Género, Estudios Transversales

## Abstract

**Objective:**

To identify factors associated with alcohol and other drug use among transgender people living in Espírito Santo.

**Methods:**

This cross-sectional study was conducted with transgender adults using a structured questionnaire. The questionnaire collected sociodemographic and health variables, as well variables on presence of depressive symptoms, quality of life, sleep health and body dissatisfaction. Substance use was assessed using the Alcohol, Smoking and Substance Involvement Screening Test (ASSIST). Multivariate linear regression models adjusted for confounding factors were analyzed, with a 5% significance level for all tests.

**Results:**

The final sample consisted of 103 transgender people with a median age of 24 years (18-44 years). The drugs with the highest scores and greatest need for intervention were tobacco (45.6%), marijuana (42.7%) and alcohol (32%). Furthermore, approximately 10% of the sample needed intervention for illicit substances. Regression analyses indicated that severity of depressive symptoms, not having undergone gender-affirming surgery, income, education, presence of comorbidities, age, quality of life and smoking were associated with increased consumption of one or more of the substances assessed (p-value<0.050 for all).

**Conclusion:**

A significant proportion of participants abused one or more of the drugs assessed. Furthermore, consumption of the substances assessed was associated with multiple social and health determinants, which act intersectionally, reinforcing the vulnerability of this population to drug use.

## Resumen

**Objetivo:**

Identificar los factores asociados al consumo de alcohol y otras drogas entre personas transgénero residentes en Espírito Santo.

**Métodos:**

Estudio transversal realizado con adultos transgénero mediante un cuestionario estructurado. Este cuestionario recopiló variables sociodemográficas y de salud, así como la presencia de síntomas depresivos, calidad de vida, salud del sueño e insatisfacción corporal. El consumo de sustancias se evaluó mediante la Prueba de Detección de Consumo de Alcohol, Tobacco y Sustancias (ASSIST). Se analizaron modelos de regresión lineal multivariante ajustados por factores de confusión, con un nivel de significancia del 5% para todas las pruebas.

**Resultados:**

La muestra final estuvo compuesta por 103 personas transgénero con una mediana de edad de 24 años (18-44 años). Las drogas con las puntuaciones más altas y mayor necesidad de intervención fueron el tabaco (45,6%), la marihuana (42,7%) y el alcohol (32%). Además, aproximadamente el 10% de la muestra requirió intervención por consumo de sustancias ilícitas. Los análisis de regresión indicaron que la gravedad de los síntomas depresivos, no haberse sometido a cirugía de afirmación de género, los ingresos, la educación, la presencia de comorbilidades, la edad, la calidad de vida y el tabaquismo se asociaron con un mayor consumo de una o más de las sustancias evaluadas (p-valor<0,050 para todos).

**Conclusión:**

Una proporción significativa de los participantes abusó de una o más de las drogas evaluadas. Además, el consumo de las sustancias evaluadas se asoció con múltiples determinantes sociales y de salud que actúan de forma interseccional, reforzando la vulnerabilidad de esta población al consumo de drogas.

**Ethical aspects**

This research respected ethical principles, having obtained the following approval data:

Research ethics committee: Universidade Federal do Espírito Santo

Opinion number: 4,431,995

Approval date: 1/12/2020

Certificate of submission for ethical appraisal: 36320620.6.0000.5060

Informed consent record: Obtained from all participants.

## Introduction 

The term transgender is neither universal nor consensual in Brazil. As such, the generic term "transgender" encompasses people whose gender identity and/or expression diverges from cultural expectations based on the biological sex assigned to them at birth^1^. These people are exposed to multiple social vulnerabilities that negatively impact their mental health and quality of life^1^^,^^2^. These negative and recurring experiences place them at substantial risk of abusing licit and illicit substances, developing anxiety, depression and non-suicidal self-harm, in addition to the risk of committing suicide^1^^,^^3^.

Despite research progress in recent years, studies have predominantly assessed alcohol and tobacco consumption among this population segment, whereby investigations into use of other substances are less frequent. A scoping review of substance use among young transgender people found that 79% of studies focused on alcohol use, while only 12% examined opioid use^4^. A history of negative healthcare experiences, such as discrimination by health professionals, barriers to accessing health and care services, as well as difficulties in obtaining legal gender recognition, are factors that can lead to use of licit and illicit substances as a coping mechanism^4^. Although limited, existing studies indicate significant differences in drug use among transgender people compared to the cisgender population^5^^,^^6^.

Barriers to accessing health services and discrimination contribute to self-medication and hinder adequate care for this population^7^. In specific contexts, such as sex work and unsupervised hormone use, the risk of problematic drug use can be even greater. It should be noted that Brazil has one of the highest numbers of homicides of transgender people in the world^8^ and, in addition to explicit violence, they still face barriers to accessing basic public services^7^.

Therefore, a more in-depth study of the unique characteristics and experiences of this population segment is necessary to identify factors associated with use of legal and illegal substances. In this context, investigating drug use among transgender people is essential for broadening our understanding of their health needs and informing interventions based on equity, harm reduction and human rights promotion. As such, this study aims to identify factors associated with use of alcohol and other drugs among transgender people.

## Methods 

Design

This is a descriptive cross-sectional study involving transgender people living in the state of Espírito Santo.

Participants and sample 

The invitation to participate in the study was made via an electronic link on the Google Forms platform, distributed through digital media (Instagram, Facebook, WhatsApp and email) to associations focused on the LGBTQIAPN+ segment and to participants attending the Gender Diversity Outpatient Clinic at the Cassiano Antônio Moraes University Hospital. The study included those who selected the option "transgender man, transgender woman, or non-binary gender" as their gender identity. Those under 18 years of age and those not residing in Espírito Santo were excluded from the analyses. The final sample, calculated using G-Power software, was representative of the state's transgender population with a 95% Confidence Interval (95%CI) and a 9.5% sampling error^9^. These calculations were based on the prevalence of transgender/non-binary people in Brazil (2%)^10^ and the number of inhabitants in the state^11^.

Data collection 

Volunteers completed the questionnaires anonymously from July 2022 to July 2024, with the aim of achieving greater honesty in their responses and reducing the bias of socially desirable responses. Participants who had any diagnosis or indications of comorbidities or distress were referred for care by a multidisciplinary team at the referral Health Service.

Sociodemographic variables 

Age was determined in years and subsequently categorized according to the median (<24 and ≥24 years). Race/skin color was self-reported according to the Brazilian Institute of Geography and Statistics criteria^12^ and categorized as "White" and "Black and/or Brown" (Brazilian mixed race). Sex assigned at birth was categorized as "male" or "female." Education level was categorized as "complete elementary school," "complete high school", or "complete higher education/master's/doctorate". Marital status was classified as "stable union" or "single". Occupation was categorized as "student", "employed" or "unemployed". Family income (per capita) was categorized as <1 minimum wage, 1 to 2 minimum wages, and >2 minimum wages according to the amounts ​​in effect in the respective years. Place of residence was categorized as "state interior" or "state capital"^13^.

Health variables 

Presence of comorbidities was self-reported and categorized as "yes" or "no". Surgery history was assessed by asking whether any gender-affirming surgery had been performed. Medical follow-up was categorized as "yes" or "no". Follow-up with a healthcare professional other than a doctor was determined by their seeing a nutritionist, speech therapist, psychologist/psychiatrist, or more than one health professional.

Body mass was self-reported taking weight in kilograms and height in meters. In this way, nutritional status was determined using the Body Mass Index (BMI) according to World Health Organization (WHO) criteria^14^ and categorized as "underweight", "normal weight" or "overweight".

Having experienced transphobia was determined via self-reporting and categorized as "yes" or "no". Hormone therapy was self-reported as "yes" or "no", and duration of hormone therapy was classified as <2 years and ≥2 years.

Self-image perception was assessed using the Brazilian Figure Scales for Adults and Children^15^. Self-perception of feelings and depression was obtained using the Beck Inventory^16^. This inventory consists of 21 groups of statements and is graded from 0 to 3 points, with statements about how the participant has been feeling in the last week. The results are expressed in points and classified into four groups: (a) 0-11, no depression; (b) 12-19, mild depression; (c) 20-35, moderate depression; (d) 36-63, severe depression. Subsequently, these groups were categorized as "no depression (score 0-11)" and "presence of depression (score ≥12)".

Quality of life was assessed using the Short-Form 6-Dimensions (SF-6D) questionnaire to describe health states by evaluating six dimensions: physical functioning; social functioning; role limitations; pain; mental health; and vitality. Scores range from 0 to 1, with 0 representing poorer health and 1 indicating better health^17^, which is then categorized according to the median. Finally, sleep quality was also assessed using an adapted and validated version of the PROMIS scale^18^ to measure sleep disorders, whereby the results were was dichotomized into "no insomnia" (score <25) or "insomnia" (score ≥25).

Outcome variable 

The alcohol and other drug use variable was obtained by administering the Alcohol, Smoking and Substance Involvement Screening Test (ASSIST)^6^, used due to the lack of validated scales for the transgender population. The ASSIST test was developed by the WHO and validated for the Brazilian population as a screening questionnaire consisting of eight questions. Those with a score ≤10 for alcohol and ≤3 for other drugs were categorized as "no intervention needed". Conversely, those with a score ≥4 for drugs and ≥11 for alcohol were categorized as "intervention needed"^19^.

Data analysis 

Data normality was verified using the Shapiro-Wilk test. In order to characterize the sample, continuous variables were expressed as mean, standard deviation and median (minimum-maximum), while categorical variables were expressed as percentages. Missing data were analyzed using the expectation-maximization method to avoid bias arising from exclusions due to incomplete data.

Differences between gender identity categories were explored using the chi-square and Fisher's exact tests to reduce the risk of type I or II errors. One-way analysis of variance and the Kruskal-Wallis test were used to assess alcohol and other drug use scores according to sociodemographic and health variables. Multivariate linear regression analysis was used to determine the influence of the independent variables on alcohol and other drug use. The sociodemographic and health variables used in the linear regression were included because they are potential confounders. This approach allowed adjustment for the effects of the independent variables, controlling for the impact of factors that could distort association between gender identity and licit and illicit substance use. All variables with a p-value<0.200 in the bivariate analyses were included. Unadjusted and adjusted models for age, race/skin color, education, income, gender identity, and marital status were analyzed. Statistical analyses were performed using SPSS v.22 with a 5% significance level.

## Results 

The final sample included 103 transgender people, including 64 transgender men (62.1%), 21 transgender women (20.3%), and 18 non-binary individuals (17.4%) (Table 1). The participants' median age was 24 years (18-44 years), with no difference between gender identities. There was a higher proportion of people of Black or Brown race/skin color (60.2%), students and/or unemployed (52.4%), residing in the state capital (79.6%), single (79.2%), and with high school and/or elementary education (80.6%). Furthermore, 44.9% of individuals had a per capita income between 1 and 2 minimum wages, 55.3% were undergoing gender-affirming hormone therapy, of whom only 32.3% had been on hormone therapy for a period equal to or greater than 2 years, and 35.9% used hormones without a prescription. Regarding nutritional status, approximately half of the sample (49.5%) was classified as overweight according to the BMI.

The scores for licit and illicit substances assessed indicated that the drugs with the greatest need for intervention were tobacco (45.6%), marijuana (42.7%) and alcohol (32%) (Table 2). Furthermore, approximately 10% of the sample required intervention for use of cocaine, amphetamines, hallucinogens and hypnotics. When the scores were compared across gender identities, a higher consumption of amphetamines and inhalants was found among non-binary people when compared to the other categories (p-value 0.013). The same occurred for the consumption of hallucinogens, which was higher among non-binary people compared to transgender women (p-value 0.022). No statistically significant differences were observed for the other substances.

In order to screen the transgender population for sociodemographic and health variables associated with alcohol and other drug use, a table (Supplementary Table 1) was created to present substance scores according to the categories of these variables. The results revealed a large number of p-values>​0.200, demonstrating the relationship between substance use and the variables studied. Multivariate linear regression analyses were performed using these data.

The multivariate linear regression analyses adjusted for possible confounding factors indicated that consumption of licit and illicit substances is associated with: depressive symptoms, quality of life, smoking, education, income, comorbidities, gender-affirming surgery, biological sex assigned at birth, age, number of residents and occupation (p-value<0.050 for all) (Table 3).

Regarding alcohol consumption, the adjusted model showed that quality of life (β 16.056; 95%CI 2.946; 29.166), smoking (β 3.969; 95%CI 6.199; 20.027), being a student and/or unemployed (β 3.773; 95%CI 1.181; 6.365), in addition to the depressive symptoms score (β 0.186; 95%CI 0.073; 0.299), were associated with increased consumption of this substance (Table 3). Similarly, being a smoker (β 3.832; 95%CI 1.217; 6.446), not having undergone gender-affirmation surgery (β 2.041; 95%CI 0.642; 3.440) and the depressive symptoms score (β 0.114; 95%CI 0.007; 0.220) were associated with increased marijuana use by study participants.

Smoking was associated with age on its own, even after other adjustments, whereby increasing age was related to higher scores for tobacco use (β 0.352; 95%CI 0.072; 0.632). Smoking was also associated with higher consumption of cocaine (β 2.041; 95%CI 0.642; 3.440), hallucinogens (β 1.707; 95%CI 0.246; 2.206) and amphetamines (β 1.022; 95%CI 0.088; 1.957). Regarding the latter substance, income below the minimum wage showed negative association (β -0.590; 95%CI -1.268; -0.050) (Table 3).

Biological sex assigned at birth was associated differently depending on the substance assessed. Being assigned female biological sex at birth was positively associated with inhalant use (β 0.572; 95% CI 0.106; 1.039) and negatively associated with opioid use (β -1.211; 95% CI -2.240; -0.181).

Regarding consumption of hypnotics, we found that the score for depressive symptoms was positively associated (β 0.086; 95%CI 0.014; 0.158), while being a student and/or unemployed showed an inverse association (β -1.707; 95%CI -3.340; -0.075). Similarly, living alone was negatively related to consumption of hallucinogens (β -1.231; 95%CI -2.206; -0.265).

Furthermore, having undergone gender-affirming surgery (β -1.746; 95%CI -2.841; -0.651) or having comorbidities (β -1.192; 95%CI -2.080) were inversely associated with consumption of other substances, while those with a lower level of education (β 1.255; 95%CI 0.294; 2.217) were associated with higher consumption of other substances (Table 3).

**Table 1:** Participant characteristics, both total and by gender identity. Espírito Santo, 2022-2024 (n=103)

Variáveis	Total n (%)	Transgender woman n (%)	Transgender man n (%)	Non-binary n (%)	p-valor
Total	103 (100.0)	21 (20.3)	64 (62.1)	18 (17.4)	
Age					0.958
<24 years	44 (42.7)	10 (45.5)	27 (41.5)	8 (43.8)	
≥24 years	59 (57.3)	11 (54.5)	37 (58.5)	10 (56.3)	
Race/skin color					0.360
White (a)	41 (39.8)	6 (28.6)	29 (45.3)	6 (33.3)	
Black and/or Brown	62 (60.2)	15 (71.4)	35 (54.7)	12(66.7)	
Occupation					0.057
Student and/or unemployed	54 (52.4)	9 (42.9)	31 (48.4)	14(77.8)	
Employed	49 (47.6)	12 (57.1)	33 (51.6)	4 (22.2)	
Schooling					0.047
Complete high school or elementary education	83 (80.6)	13 (61.9)	55 (85.9)	15 (83.3)	
Higher education/master's/doctorate	20 (19.4)	8 (38.1)	9 (14.1)	3 (16.7)	
Income (minimum wages)^a^					0.001
<1	17 (17.3)	3 (15.0)	7 (11.3)	7 (43.8)	
1-2	44 (44.9)	4 (20.0)	35 (56.5)	5 (31.3)	
>2	37 (37.8)	13 (65.0)	20 (32.3)	4 (25.0)	
Marital status					0.081
Single	81 (79.2)	19 (90.5)	45 (70.4)	17 (94.4)	
Stable union	22 (21.4)	2 (9.5)	19 (29.7)	1 (5.6)	
City					0.508
State capital	82 (79.6)	15 (71.4)	53 (82.8)	14 (77.8)	
State interior	21 (20.4)	6 (28.6)	11 (17.2)	4 (22.2)	
Hormone therapy					0.004
Yes	57 (55.3)	15 (71.4)	38 (59.4)	4 (22.2)	
No	46 (44.7)	6 (28.6)	26 (40.6)	14 (77.8)	
Hormone therapy without prescription					<0.001
Yes	37 (35.9)	15 (71.4)	19 (29.7)	3 (16.7)	
No	66 (64.1)	6 (28.6)	45 (70.3)	15 (83.3)	
Duration of hormone therapy (years)					0.028
<2	21 (21.2)	5 (25)	13 (21.3)	3 (16.7)	
≥2	32 (32.3)	9 (45.0)	22 (36.1)	1 (5.6)	
Not on therapy	46 (46.5)	6 (30.0)	26 (42.6)	14 (77.8)	
Gender-affirming surgery^b^					0.523
Yes	15 (14.7)	4 (19.0)	10 (15.6)	1 (5.9)	
No	87 (85.3)	17 (81.0)	54 (84.4	16 (94.1)	
Experience of transphobia					0.069
Yes	91 (88.3)	21 (100.0)	53 (82.8)	17 (94.4)	
No	12 (11.7)	0 (0.0)	11 (17.2)	1 (5.6)	
Body mass index					0.106
Low weight	9 (8.7)	3 (14.3)	3 (4.7)	3 (16.7)	
Normal weight	43 (41.7)	12 (57.1)	24 (37.5)	7 (38.9)	
Overweight	51 (49.5)	6 (28.6)	37 (57.8)	8 (44.4)	
Presence of comorbidity					0.946
Yes	25 (24.3)	5 (23.8)	15 (23.4)	5 (27.8)	
No	78 (75.7)	16 (76.2)	49 (76.6)	13 (72.2)	
Depression					0.523
Yes	75 (73.0)	17 (79.2)	44 (69.2)	14 (80)	
No	28 (27.0)	4 (20.8)	20 (30.8)	4 (20)	
Sleep health					0.615
Absence of insomnia	35 (34.0)	9 (40.9)	20 (30.8)	7 (37.5)	
Presence of insomnia	68 (76.0)	12 (59.1)	44 (69.2)	11 (62.5)	
Quality of life					0.350
Below median	51 (49.5)	9 (36.4)	34 (53.8)	8 (43.8)	
Above or equal to median	52 (50.5)	16 (64.6)	30 (46.2)	10 (56.2)	

a n=98; ^b^n=101.

**Table 2:** Score (median: minimum and maximum) and classification of need for intervention (%) for consumption of alcohol and other drugs in the total population and by gender identity. Espírito Santo, 2022-2024 (n=103)

Variables	Total n (%)	Transgender woman n (%)	Transgender man n (%)	Non-binary n (%)	p-value
Alcohol (score)	7 (0;29)	6 (0;24)	6.5 (0;29)	7.5 (0;27)	0.902
Alcohol (classification)					0.473
Intervention not needed	70 (68.0)	15 (71.4)	45 (70.3)	10 (55.6)	
Intervention needed	33 (32)	6 (28.6)	19 (29.7)	8 (44.4)	
Tobacco (score)	3 (0;26)	3 (0;22)	3 (0;26)	5.5 (0;25)	0.446
Tobacco (classification)					0.586
Intervention not needed	56 (54.4)	13 (61.9)	35 (54.7)	8 (44.4)	
Intervention needed	47 (45.6)	8 (38.1)	29 (45.3)	10 (55.6)	
Marijuana (score)	2 (0;30)	5 (0;14)	1 (0;30)	2 (0;23)	0.729
Marijuana (classification)					0.341
Intervention not needed	59 (57.3)	9 (42.9)	39 (60.9)	11 (61.1)	
Intervention needed	44 (42.7)	12 (57.1)	25 (39.1)	7 (38.9)	
Cocaine (score)	0 (0;20)	0 (0;20)	0 (0;20)	0 (0;12)	0.437
Cocaine (classification)					0.424
Intervention not needed	94 (91.3)	19 (90.5)	60 (93.8)	15 (83.3)	
Intervention needed	9 (8.7)	2 (9.5)	4 (6.3)	3 (16.7)	
Amphetamines (score)	0 (0;15)	0 (0;2)	0 (0;10)	0 (0;15)^a,b^	0.013
Amphetamines (classification)					0.126
Intervention not needed	96 (93.2)	21 (100)	60 (93.8)	15 (83.3)	
Intervention needed	7 (6.8)	0 (0)	4 (6.3)	3 (16.7)	
Inhalants (score)	0 (0;10)	0 (0;6)	0 (0;9)	0 (0;10)^a,b^	0.011
Inhalants (classification)					0.350
Intervention not needed	98 (95.1)	20 (95.2)	62 (96.9)	16 (88.9)	
Intervention needed	5 (4.9)	1 (4.8)	2 (3.1)	2 (11.1)	
Hypnotics (score)	0 (0;22)	0 (0;18)	0 (0;22)	0 (0;17)	0.434
Hypnotics (classification)					0.554
Intervention not needed	89 (86.4)	19 (90.5)	56 (87.5)	14 (76.8)	
Intervention needed	14 (13.6)	2 (9.5)	8 (12.5)	4 (22.2)	
Hallucinogens (score)	0 (0;13)	0 (0;5)	0 (0;13)	0 (0;10)^a^	0.022
Hallucinogens (classification)					0.476
Intervention not needed	94 (91.3)	20 (95.2)	59 (92.2)	15 (83.3)	
Intervention needed	9 (8.7)	1 (4.9)	5 (7.8)	3 (16.7)	
Opioids (score)	0 (0;13)	0 (0;12)	0 (0;7)	0 (0;13)	0.275
Opioids (classification)					0.559
Intervention not needed	99 (96.1)	20 (95.2)	62 (96.9)	17 (94.4)	
Intervention needed	4 (3.9)	1 (4.8)	2 (3.1)	1 (5.6)	
Other drugs (score)	0 (0;17)	0 (0;17)	0 (0;5)	0 (0;3)	0.535
Other drugs (classification)					0,183
Intervention not needed	100 (97,1)	19 (90,5)	63 (98,4)	18 (100)	
Intervention needed	3 (2,9)	2 (9,5)	1 (1,6)	(0)	

a Compared to transgender women; ^b^ Compared to transgender men.

**Table 3:** Unadjusted and adjusted coefficients (β) and 95% confidence intervals (95%) between the score for alcohol and other drug consumption and independent variables. Espírito Santo, 2022-2024 (n=103)

Variables	Unadjusted β (95%)	p-value	Adjusted β (95%)	p-value
Alcohol				
Quality of life	16.786 (3.968; 29.604)	0.011	16.056 (2.946; 29.166)	0.017
Tobacco smoking	4.335 (1.666; 7.003)	0.002	3.969 (6.199; 20.027)	0.004
Student and/or unemployed	3.612 (1.085; 6.139)	0.006	3.773 (1.181; 6.365)	0.005
Depressive symptoms score	0.179 (0.069; 0.289)	0.002	0.186 (0.073; 0.299)	0.002
Tobacco				
Age	0.337 (0.057; 0.616)	0.019	0.352 (0.072; 0.632)	0.014
Marijuana				
Tobacco smoking	4.715 (2.084; 7.346)	0.001	3.832 (1.217; 6.446)	0.005
Not having undergone gender-affirming surgery	3.438 (0.003; 6.874)	0.050	2.041 (0.642; 3.440)	0.040
Depressive symptoms score	0.115 (0.008; 0.223)	0.036	0.114 (0.007; 0.220)	0.037
Cocaine				
Being a smoker	1.990 (0.649; 3.331)	0.004	2.041 (0.642; 3.440)	0.005
Amphetamines				
Being a smoker	1.022 (0.088; 1.957)	0.032	1.022 (0.088; 1.957)	0.032
<1 minimum wage	-0.659 (-1.268; 0.050)	0.034	-0.590 (-1.260; 0.051)	0.034
Inhalants				
Female biological sex	0.572 (0.106; 1.039)	0.017	0.572 (0.106; 1.039)	0.017
Hypnotics				
Depressive symptoms score	0.086 (0.014; 0.158)	0.020	0.086 (0.014; 0.158)	0.020
Student and/or unemployed	-1.707 (-3.340; -0.075)	0.041	-1.707 (-3.340; -0.075)	0.041
Hallucinogens				
Being a smoker	1.716 (0.725; 2.706)	0.001	1.707 (0.246; 2.206)	0.002
Living alone	-1.224 (-2.157; 0.291)	0.011	-1.231 (-2.206; -0.265)	0.014
Opioids				
Female biological sex	-1.496 (-2.563; -0.430)	0.006	-1.211 (-2.240; -0.181)	0.022
Other substances				
Having undergone gender-affirming surgery	-1.513 (-2.622; -0.403)	0.008	-1.746 (-2.841; -0.651)	0.002
Presence of comorbidities	-1.209 (-2.121; -0.297)	0.010	-1.192 (-2.080; -0.304)	0.009
Complete elementary education/complete high school education	1.211 (0.223; 2.198)	0.017	1.255 (0.294; 2.217)	0.011

## Discussion

The results of this study showed that the severity of depressive symptoms, not having undergone gender-affirming surgery, income, education, comorbidities, age, quality of life and smoking were associated with increased consumption of one or more of the licit and illicit substances assessed. Among these, the most frequently consumed were tobacco, marijuana and alcohol. Additionally, all other substances investigated presented scores indicative of the need for intervention, demonstrating a pattern of potentially abusive substance use. These results corroborate data from a longitudinal cohort study that monitored young people from sexual and gender minorities in the United States whose biological sex was assigned as male at birth^20^. In that study, approximately 4.7% of participants were at high risk for alcohol use disorder, 14% had scores consistent with possible cannabis use disorder, 9.8% reported stimulant use in the past six months, 7% reported nightclub drug use, and 10.7% reported use of other illicit drugs^20^.

A systematic review involving 25,233 transgender adults demonstrated that stigma and violence against these individuals are common and that the risk of experiencing this type of discrimination is well established and has been associated with engagement in health-risk behaviors, such as use of licit and illicit substances as a means of coping^5^. The concept of minority stress suggests that members of marginalized social groups experience stress overload, which may contribute to maladaptive coping behaviors, such as the use of psychoactive substances^21^. In this context, a Dutch study of transgender youth demonstrated that high rates of drug use among gender minorities are often explained by their experiences with minority stressors^22^. This model argues that social minorities experience specific stressors in addition to everyday stressors^23^.

Another important study involving transgender adults from Massachusetts and Rhode Island revealed that 60.5% of participants had had at least one experience of physical or sexual abuse in childhood, 87.4% had experienced discrimination based on gender identity, and 30.5% reported a history of intimate partner violence^24^. Analyses also revealed that high scores for discrimination against transgender people were positively associated with substance use^24^. The results of our study corroborate the findings of the aforementioned articles, as many socioeconomic and health variables were associated with alcohol and other drug use.

In Brazil, few studies have addressed alcohol and other drug use among this population. One such study, conducted in five Brazilian regions, found that marijuana, sedatives and tranquilizers, as well as cocaine, antihistamines and hookah were the most commonly used substances among transgender individuals^25^. According to the authors, the main factors associated with substance use were discrimination and unstable family environment. Another study, conducted with Brazilian transgender women, found a greater propensity to use illicit drugs before or during sex, with the aim of enhancing, disinhibiting or facilitating the sexual experience^26^. These relationships demonstrate the intersectionality of specific vulnerabilities within this population segment. A wide-ranging investigative perspective is necessary, since intersectionality seeks to grasp the complexity of identities and social inequalities through integration with the main axes of social differentiation, such as sex/gender, class, race/skin color, ethnicity, age, disability, sexual orientation, among others^27^.

Previous studies have found that alcohol is one of the most prevalent substances among the transgender population^28^^,^^29^, and in 2020, 60.4% of this population reported regular consumption^30^. The stigma this population faces and their experiences of prejudice put them at greater risk of problematic alcohol use^31^. In line with previous studies, this study identified a linear association between the severity of depressive symptoms and increased consumption of alcohol, marijuana and hypnotics. Consumption of licit and illicit substances is closely related to the emergence or worsening of depressive symptoms, due to their neurobiological and psychosocial effects^32^. From a pathophysiological perspective, substances such as alcohol act by modulating neurotransmitters, initially producing feelings of euphoria or relaxation and, later, with repeated or excessive use, generating changes in neuronal plasticity and dysfunctions in the brain reward and mood circuits^32^^,^^33^. Such changes can precipitate or intensify depressive symptoms, especially in individuals exposed to psychosocial stressors^34^. Furthermore, drug use often emerges as a strategy for temporary relief from depressive symptoms, creating a two-way cycle in which consumption maintains and worsens depressive symptoms, hindering clinical recovery and increasing the risk of psychiatric and social complications^32^^,^^34^.

Another relevant finding of this study was the positive association between not having undergone gender-affirming surgery and use of marijuana and other drugs. A North American study of 27,715 transgender people demonstrated that undergoing gender-affirming surgery was associated with reduced likelihood of psychological distress and smoking^35^. However, after adjustments, that study found no association between undergoing gender-affirming surgery and lifetime alcohol use and/or smoking. Be that as it may, other studies demonstrate that transgender people who are unable to access gender-affirming surgery may have higher prevalence of drug use, due to both psychological and social factors^36^^,^^37^. Unresolved gender dysphoria, coupled with chronic distress due to incongruence between body and gender identity, significantly contributes to symptoms of anxiety, depression and stress, which are conditions often related to use of licit and illicit substances^37^^,^^38^. Furthermore, structural barriers hinder access to gender-affirming surgery, increasing feelings of marginalization and social exclusion among this population^36^^,^^39^. Such circumstances can foster risk behaviors, including use of illicit drugs, often as an attempt at temporary emotional relief^39^. These results reinforce the specific health characteristics of this population and how they can impact their mental health.

This study has several limitations. First, because it is cross-sectional, it does not allow for the establishment of causal relationships between events, although careful analysis was adopted to reduce bias. The second limitation concerns the predominance of transgender men in the sample, which reflects their greater demand for outpatient care. Finally, the third limitation is the use of instruments validated for cisgender individuals. Such instruments, based on cisnormative conceptions, do not address the specific experiences of transgender people nor the social and cultural complexities that impact this population. The scarcity of psychometric instruments suitable for this group represents a significant gap for the scientific community.

Despite these limitations, the use of the ASSIST questionnaire (screening test for alcohol, tobacco and substance use) stands out, as it is validated and easy to administer in primary care. In this sense, the results indicate the need for routine screening for mood disorders and substance use in vulnerable populations. Furthermore, they highlight the urgent need for longitudinal studies that elucidate the causal mechanisms between gender dysphoria, mental health and drug use, as well as clinical trials that test specific interventions for this population.

In summary, the results reinforce the high vulnerability of transgender people to use of substances such as tobacco, marijuana and alcohol. Socioeconomic and health variables were associated with use of alcohol and other drugs, highlighting the influence of social determinants. The urgent need to eliminate barriers to access to gender-affirming surgery is also highlighted, as it can reduce psychological distress and risk behaviors. Finally, it is clear that substance use in this group stems from the intersection of structural, emotional and clinical factors. Thus, the data reinforce the importance of intersectoral public policies and integrated, inclusive care strategies that are sensitive to the inequalities faced by the transgender population.

## Data Availability

The data are protected for ethical reasons, however they may be made available upon request.

## References

[1] 1. Ugueto AM, Lucassen MFG. Differences in suicidality and psychological symptoms between sexual and gender minority youth compared to hetero sexual and cisgender youth in a psychiatric hospital. Child Youth Serv Rev. 2022;138:106504.

[2] 2. Coswosck KHC, Moreira JA, Navarro JHN, et al. Factors associated with poor quality of life of transgender people. Discov Soc Sci Health. 2022;2:17.

[3] 3. Hanna B, Desai R, Parekh T, Guirguis E, Kumar G, Sachdeva R. Psychiatric disorders in the U.S. transgender population. Ann Epidemiol. 2019;39:1-7.e1. 10.1016/j.annepidem.2019.09.009 31679894

[4] 4. Kidd JD, Kidd JD, Jackman KB, Wolff M, Veldhuis CB, Hughes TL. Risk and Protective Factors for Substance Use Among Sexual and Gender Minority Youth: a Scoping Review. Curr Addict Rep. 2018;5(2):158-3. 10.1007/s40429-018-0196-9 PMC6214200 30393591

[5] 5. Connolly D, Gilchrist G. Prevalence and correlates of substance use among Transgender adults: A systematic review. Addict Behav. 2020;111:106544. 10.1016/j.addbeh.2020.106544 32717497

[6] 6. Frost MC, Blosnich JR, Lehavot K, Chen JA, Rubinsky AD, Glass JE, Williams EC. Disparities in Documented Drug Use Disorders Between Transgender and Cisgender U.S. Veterans Health Administration Patients. J Addict Med. 2021;15(4):334-40. 10.1097/ADM.0000000000000769 PMC8384140 33252409

[7] 7. Coelho DRA, Ferreira ALN, Vieira WF, Keuroghlian AS, Reisner SL. Mental Health Outcomes Among *Travestis* and Transgender Women in Brazil: A Literature Review and a Call to Action for Public Health Policies. Int J Environ Res Public Health. 2025; 22(7):977. 10.3390/ijerph22070977 PMC12294298 40724043

[8] 8. Costa AB, Da Rosa Filho HT, Pase PF, Fontanari AMV, Catelan RF, Mueller A, et al. Healthcare needs of and Access barriers for brazilian transgender and gender diverse people. J Immigr Minor Health. 2016;20(1):115-23. 10.1007/s10903-016-0527-7 27804013

[9] 9. Faul F, Erdfelder E, Lang AG, Buchner A. G*Power 3: a flexible statistical Power analysis program for the social, behavioral, and biomedical sciences. Behav Res Methods. 2007 May;39(2):175-91. 10.3758/bf03193146 17695343

[10] 10. Spizzirri G, Eufrásio R, Lima, MCP. et al. Proportion of people identified as transgender and non-binary gender in Brazil. Sci Rep. 2021; 1(11):2240-7. 10.1038/s41598-021-81411-4 PMC7838397 33500432

[11] 11. Instituto Brasileiro de Geografia e Estatística (IBGE) [Internet]. Densidade demográfica: Censo 2022: População e Domicílios - Primeiros Resultados - Atualizado em 22/12/2023. [cited 2025 Jul 8]. Available from: https://www.ibge.gov.br/cidades-e-estados/es.html.

[12] 12. Instituto Brasileiro de Geografia e Estatística [Internet]. Características Étnico-Raciais da População Classificações e Identidades 2011. [cited 2025 Oct 01]. Available from: Available from: https://www.ibge.gov.br/ estatisticas/sociais/populacao/9372-caracteristicas-etnico-raciais-da-populacao.html .

[13] 13. Regiões de Saúde do ES, do PDR 2020 estabelecidos na Resolução CIB 153, 2020 SESA/ES. [Internet] [cited 2025 Oct 01]. Available from: https://saude.es.gov.br/Media/sesa/CIB/Resolução%20CIB%20 153-2020%20-%20PDR%20ES%202020.pdf

[14] 14. World Health Organization. WHO child growth standards: length/height-for-age, weight-for-age, weight-for-length, weight-for-height and body mass index-for-age: methods and development [Internet]. World Health Organization. 2006. [cited 2025 Oct 01]. Available from: Available from: https://www.who.int/publications/i/ item/924154693X .

[15] 15. Kakeshita IS, Silva AI. P, Zanatta DP, Almeida SS. Construção e fidedignidade teste-reteste de escalas de silhuetas brasileiras para adultos e crianças. Psic.: Teor. e Pesq. 2009;25(2):263-70.

[16] 16. Cunha JA. BDI BAI BHS BSI. Manual da Versão em Português das escalas de Beck [Internet]. São Paulo: Casa do Psicólogo; 2001. [cited 2025 Jun 30]. Available from: Available from: https://pt.scribd.com/ document/426714190/Manual-Escalas-Beck

[17] 17. Berwanger DD, Filippin LI, Saldanha RP. Questionário SF-6D Brasil: Modelos de construção e aplicações em economia da saúde rev. Assoc. Med. Bras. 2010;56(4):409-14.

[18] 18. Costa ZMS, Pinto RMC, Mendonça TMS, et al. Validação brasileira dos bancos de itens Distúrbio do Sono e Distúrbio da Vigília do Patient-Reported Outcomes Measurement Information System (PROMIS). Cad Saude Publica. 2020;36(6):00228519. 10.1590/0102-311X00228519 32520128

[19] 19. Henrique IF, De Micheli D, Lacerda RB, Lacerda LA, Formigoni ML. Validation of the Brazilian version of Alcohol, Smoking and Substance Involvement Screening Test (ASSIST). Rev Assoc Med Bras. 2004;50(2):199-206. 10.1590/s0104-42302004000200039 15286871

[20] 20. Newcomb ME; Hill R, Buehler K, Ryan DT, Whitton SW, Mustanski B. High Burden of Mental Health Problems, Substance Use, Violence, and Related Psychosocial Factors in Transgender, Non-Binary, and Gender Diverse Youth and Young Adults. Arch Sex Behav. 2020;49(2):645-59. 10.1007/s10508-019-01533-9 PMC7018588 31485801

[21] 21. Gilbert PA, Pass LE, Keuroghlian AS, Greenfield TK, Reisner SL. Alcohol research with transgender populations: A systematic review and recommendations to strengthen future studies. Drug Alcohol Depend. 2018;186:138-46. 10.1016/j.drugalcdep.2018.01.016 PMC5911250 29571076

[22] 22. Kiekens WJ, Baams L, Veenstra R. Differences by sex and gender in the association between minority stress and alcohol use among sexual and gender minority youth: A daily diary study. Soc Sci Med. 2022; 294(114679):1-10. 10.1016/j.socscimed.2021.114679 PMC7612808 35030397

[23] 23. Paveltchuk FDO, Borsa JC. A teoria do estresse de minoria em lésbicas, gays e bissexuais. Rev. SPAGESP. 2020;21(2):41-4.

[24] 24. Wolfe HL, Biello KB, Reisner SL, Mimiaga M, Cahill S, Hughto JMW. Transgender-related Discrimination and Substance Use, Substance Use Disorder and Treatment History among Transgender Adults. Drug Alcohol Depend. 2021;223:108711. 10.1016/j.drugalcdep.2021.108711 PMC8114322 33866073

[25] 25. Fontanari AMV, Pase PF, Churchill S, Solla BMB, Schwarzd K, Schneidera MA, et al. Dealing with gender-related and general stress: Substance use among Brazilian transgender youth. Addict Behav Rep. 2019;(9):100166. 10.1016/j.abrep.2019.100166 PMC6542738 31193732

[26] 26. Jalil EM, Torres TS, Pereira CCA, Farias A, Brito JDU, Lacerda M, et al. High rates of sexualized drug use or chemsex among Brazilian transgender women and young sexual and gender minorities. Int J Environ Res Public Health. 2022;19(3):1704. 10.3390/ijerph19031704 PMC8835457 35162728

[27] 27. Bilge S. Théorisations féministes de l’intersectionnalité. [Internet]. Diogène 2009;225(1):70-8. [cited 2025 Jun 30]. Available from: Available from: https://shs.cairn.info/revue-diogene-2009-1-page-70?lang=fr

[28] 28. Bradford J, Reisner SL, Honnold JA, et al. Experiences of transgender-related discrimination and implications for health: results from the Virginia Transgender Health Initiative Study. Am J Public Health. 2013;103(10):1820-1829. 10.2105/AJPH.2012.300796 PMC3780721 23153142

[29] 29. Hughto JMW, Quinn EK, Dunbar MS, et al. Prevalence and Co-occurrence of Alcohol, Nicotine, and Other Substance Use Disorder Diagnoses Among US Transgender and Cisgender Adults. JAMA Netw Open. 2021;4(2):2036512. 10.1001/jamanetworkopen.2020.36512 PMC7862992 33538824

[30] 30. Kcomt L, Evans-Polce RJ, Boyd CJ, McCabe SE. Association of transphobic discrimination and alcohol misuse among transgender adults: Results from the U.S. Transgender Survey. Drug and Alcohol Depend. 2020;215:10822. 10.1016/j.drugalcdep.2020.108223 PMC7502497 32777693

[31] 31. Kiekens WJ, Baams L, Veenstra R. Differences by sex and gender in the association between minority stress and alcohol use among sexual and gender minority youth: A daily diary study. Soc Sci Med. 2022;294:114679. 10.1016/j.socscimed.2021.114679 PMC7612808 35030397

[32] 32. Boden JM, Fergusson DM. Alcohol and depression. Addiction. 2011;106(5):906-14. 10.1111/j.1360-0443.2010.03351.x 21382111

[33] 33. Koob GF, Volkow ND. Neurobiology of addiction: a neurocircuitry analysis. Lancet Psychiatry. 2016;3(8):760-73. 10.1016/S2215-0366(16)00104-8 PMC6135092 27475769

[34] 34. Volkow ND, Morales M. The Brain on Drugs: From Reward to Addiction. Cell. 2015;162(4):712-5. 10.1016/j.cell.2015.07.046 26276628

[35] 35. Almazan AN, Keuroghlian AS. Association Between Gender-Affirming Surgeries and Mental Health Outcomes. JAMA Surg. 2021;156(7):611-8. 10.1001/jamasurg.2021.0952 PMC8082431 33909023

[36] 36. Reisner SL, Poteat T, Keatley J, et al. Global health burden and needs of transgender populations: a review. Lancet. 2016;388(10042):412-36. 10.1016/S0140-6736(16)00684-X PMC7035595 27323919

[37] 37. Bockting WO, Miner MH, Romine RES, et al. Stigma, Mental Health, and Resilience in an Online Sample of the US Transgender Population. Am J Public Health. 2013;103(5):943-51. 10.2105/AJPH.2013.301241 PMC3698807 23488522

[38] 38. Hughto JMW, Reisner SL, Pachankis JE. Transgender stigma and health: A critical review of stigma determinants, mechanisms, and interventions. Soc Sci Med. 2015;11(147):222-31. 10.1016/j.socscimed.2015.11.010 PMC4689648 26599625

[39] 39. White Hughto JM, Reisner SL. A systematic review of the effects of hormone therapy on psychological functioning and quality of life in transgender individuals. Transgend Health. 2016;1(1):21-1. 10.1089/trgh.2015.0008 PMC5010234 27595141

