# Supplementary material for: Factors associated with alcohol and other drug use among transgender people: a cross-sectional study, Espírito Santo, 2022-2024
Source: Epidemiol Serv Saude. 2026 May 16;35:e20250205. doi: 10.1590/S2237-96222026v35e20250205.en (PMC13686421; doi:10.1590/S2237-96222026v35e20250205.en)
Supplement: Supplementary file 1 [file 2237-9622-ress-35-e20250205-tabela-suplementar.pdf]

Tabela suplementar 1. Pontuação (mediana, mínima e máxima) do Consumo de álcool, tabaco, maconha, cocaína, anfetaminas, inalantes, hipnóticos, alucinógenos, opióides e outras drogas de acordo com as categorias das variáveis sociodemográficas e de saúde da amostra total da população transgênero residentes no Estado do Espírito Santo a partir da ferramenta Teste de triagem para envolvimento com álcool, tabagismo e substâncias (ASSIST), 2022-2024, n=103

Variáveis[*]	Álcool	Tabaco	Maconha	Cocaína	Anfetaminas	Inalantes	Hipnóticos	Alucinógenos	Opióides	Outras
**Sexo biológico**										
Masculino	7 (0 – 24)	1,5 (0 – 24)	5 (0 – 14)	0 (0 – 20)	0 (0 – 2)	0 (0 – 6)	0 (0 – 17)	0 (0 – 5)	0 (0 – 13)	1,5 (0 – 24)
Feminino	7 (0 – 29)	3 (0 – 26)	2 (0 – 30)	0 (0 – 20)	0 (0 – 15)	0 (0 – 10)	0 (0 – 22)	0 (0 – 13)	0 (0 – 13)	3 (0 – 26)
p-valor	0,981	0,395	0,360	0,922	0,387	0,951	0,736	0,510	0,107	0,598
**Identidade de gênero**										
Homem Trangênero	6,5 (0 – 29)	3 (0 – 26)	1 (0 – 30)	0 (0 – 20)	0 (0 – 10)	0 (0 – 10)	0 (0 – 22)	0 (0 – 13)	0 (0 – 7)	0 (0 – 5)
Mulher TRANS	6,0 (0 – 24)	3 (0 – 22)	5 (0 – 14)	0 (0 – 20)	0 (0 – 2)	0 (0 – 6)	0 (0 – 18)	0 (0 – 5)	0 (0 – 12)	0 (0 – 17)
Não binário	7,5 (0 – 27)	5,5 (0 – 25)	2 (0 – 23)	3 (0 – 12)	0 (0 – 15)[a,b]	0 (0 – 10)[a,b]	0 (0 – 17)	0 (0 – 10)[a]	0 (0 – 13)	0 (0 – 3)
p-valor	0,902	0,446	0,729	0,437	0,013	0,011	0,434	0,022	0,275	0,535
**Idade (anos)**										
<24	5,5 (0 – 24)	3 (0 – 25)	2,5 (0 – 30)	0 (0 – 22)	0 (0 – 7)	0 (0 – 3)	0 (0 – 22)	0 (0 – 13)	0 (0 – 3)	0 (0 – 3)
≥24	7 (0 – 29)	5 (0 – 26)	2 (0 – 27)	0 (0 – 22)	0 (0 – 15)	0 (0 – 10)	0 (0 – 18)	0 (0 – 12)	0 (0 – 13)	0 (0 – 17)
p-valor	0,710	0,150	0,527	0,063	0,941	0,053	0,741	0,088	0,178	0,303
**Atividade laboral**										
Empregado	8 (0 – 29)	6 (0 – 26)	2 (0 – 30)	0 (0 – 20)	0 (0 – 10)	0 (0 – 10)	0 (0 – 18)	0 (0 – 13)	0 (0 – 12)	0 (0 – 17)
Estudante e/ou desempregado	5,5 (0 – 27)	3 (0 – 25)	3 (0 – 23)	0 (0 – 12)	0 (0 – 15)	0 (0 – 10)	0 (0 – 22)	0 (0 – 10)	0 (0 – 13)	0 (0 – 3)
p-valor	0,113	0,059	0,986	0,581	0,579	0,521	0,112	0,661	0,579	0,555
**Local que vive**										
Grande Vitória	7 (0 – 27)	3 (0 – 26)	2 (0 – 30)	0 (0 – 20)	0 (0 – 15)	0 (0 – 10)	0 (0 – 22)	0 (0 – 13)	0 (0 – 13)	0 (0 – 17)
Interior	8 (0 – 29)	3 (0 – 25)	3 (0 – 15)	0 (0 – 7)	0 (0 – 5)	0 (0 – 5)	0 (0 – 7)	0 (0 – 5)	0 (0 – 5)	0 (0 – 5)
p-valor	0,761	0,986	0,796	0,571	0,231	0,843	0,294	0,753	0,791	0,684

Variáveis*	Álcool	Tabaco	Maconha	Cocaína	Anfetaminas	Inalantes	Hipnóticos	Alucinógenos	Opióides	Outras
**Estado civil**										
Solteiro	7 (0 – 27)	3 (0 – 26)	2 (0 – 30)	0 (0 – 20)	0 (0 – 15)	0 (0 – 10)	0 (0 – 22)	0 (0 – 13)	0 (0 – 13)	0 (0 – 12)
União estável	6 (0 – 29)	8 (0 – 25)	2,5 (0 – 27)	0 (0 – 3)	0 (0 – 6)	0 (0 – 3)	0 (0 – 9)	0 (0 – 12)	0 (0 – 3)	0 (0 – 17)
p-valor	0,840	0,264	0,751	0,830	0,577	0,197	0,233	0,888	0,504	0,668
**Raça/cor da pele**										
Branca	5 (0 – 26)	2 (0 – 26)	0 (0 – 18)	0 (0 – 20)	0 (0 – 15)	0 (0 – 10)	0 (0 – 21)	0 (0 – 10)	0 (0 – 13)	0 (0 – 17)
Preta ou Parda	7 (0 – 29)	4 (0 – 25)	3,5 (0 – 30)	0 (0 – 20)	0 (0 – 7)	0 (0 – 6)	0 (0 – 22)	0 (0 – 13)	0 (0 – 12)	2 (0 – 26)
p-valor	0,421	0,989	0,188	0,420	0,502	0,953	0,909	0,618	0,896	0,027
**Escolaridade**										
EM e/ou FC	6 (0 – 27)	3 (0 – 26)	2 (0 – 30)	0 (0 – 20)	0 (0 – 10)	0 (0 – 10)	0 (0 – 22)	0 (0 – 13)	0 (0 – 8)	0 (0 – 5)
ES e/ou M/D**	8 (2 – 29)	4 (0 – 25)	5 (0 – 15)	0 (0 – 12)	0 (0 – 15)	0 (0 – 10)	0 (0 – 21)	0 (0 – 10)	0 (0 – 13)	0 (0 – 17)
p-valor	0,373	0,812	0,590	0,777	0,295	0,729	0,227	0,949	0,602	0,468
**Número de pessoas na casa**										
Sozinho	9 (0 – 29)	4 (0 – 25)	3 (0 – 27)	0 (0 – 12)	0 (0 – 15)	0 (0 – 10)	0 (0 – 22)	0 (0 – 13)	0 (0 – 13)	0 (0 – 17)
>1 pessoa(s)	5 (0 – 26)	2,5 (0 – 26)	1 (0 – 30)	0 (0 – 20)	0 (0 – 10)	0 (0 – 10)	0 (0 – 21)	0 (0 – 10)	0 (0 – 12)	0 (0 – 120)
p-valor	0,039	0,123	0,095	0,637	0,027	0,156	0,729	0,030	0,055	0,099
**Renda (salário mínimo)**										
<1	7 (0 – 14)	5 (0 – 24)	4 (0 – 17)	0 (0 – 12)	0 (0 – 15)	0 (0 – 10)	0 (0 – 22)	0 (0 – 21)	0 (0 – 7)	0 (0 – 3)
1 a 2	8 (0 – 27)	5 (0 – 26)	2,5 (0 – 27)	0 (0 – 20)	0 (0 – 10)	0 (0 – 10)	0 (0 – 22)	0 (0 – 10)	0 (0 – 13)	0 (0 – 5)
>2	5 (0 – 29)	5 (0 – 25)	0 (0 – 14)	0 (0 – 20)	0 (0 – 3)	0 (0 – 6)	0 (0 – 17)	0 (0 – 13)	0 (0 – 8)	0 (0 – 17)
p-valor	0,321	0,321	0,412	0,737	0,235	0,277	0,419	0,466	0,104	0,969
**Presença de comorbidades**										
Sim	6 (0 – 24)	0 (0 – 25)	0 (0 – 27)	0 (0 – 20)	0 (0 – 6)	0 (0 – 6)	0 (0 – 21)	0 (0 – 12)	0 (0 – 12)	0 (0 – 17)
Não	7 (0 – 29)	4 (0 – 26)	3 (0 – 30)	0 (0 – 20)	0 (0 – 15)	0 (0 – 10)	0 (0 – 22)	0 (0 – 13)	0 (0 – 13)	0 (0 – 5)
p-valor	0,613	0,295	0,497	0,164	0,069	0,557	0,081	0,337	0,954	0,034

Variáveis[*]	Álcool	Tabaco	Maconha	Cocaína	Anfetaminas	Inalantes	Hipnóticos	Alucinógenos	Opióides	Outras
**Hormonização**										
Sim	6 (0 – 29)	0 (0 – 26)	3 (0 – 30)	0 (0 – 20)	0 (0 – 10)	0 (0 – 10)	0 (0 – 22)	0 (0 – 13)	0 (0 – 12)	0 (0 – 17)
Não	8 (0 – 27)	4,5 (0 – 25)	2 (0 – 24)	0 (0 – 12)	0 (0 – 15)	0 (0 – 10)	0 (0 – 17)	0 (0 – 10)	0 (0 – 13)	0 (0 – 3)
p-valor	0,201	0,195	0,748	0,761	0,922	0,613	0,870	0,367	0,310	0,867
**Procedimento cirúrgico de afirmação sexual**										
Sim	5 (0 – 26)	4 (0 – 26)	0 (0 – 10)	0 (0 – 20)	0 (0 – 10)	0 (0 – 10)	0 (0 – 18)	0 (0 – 10)	0 (0 – 12)	0 (0 – 17)
Não	7 (0 – 29)	3 (0 – 25)	3 (0 – 30)	0 (0 – 20)	0 (0 – 15)	0 (0 – 10)	0 (0 – 22)	0 (0 – 13)	0 (0 – 13)	0 (0 – 5)
p-valor	0,133	0,458	0,045	0,384	0,807	0,336	0,670	0,148	0,362	0,240
**Tabagismo**										
Sim	9,5 (0 – 29)	15 (0 – 26)	5,5 (0 – 30)	0 (0 – 20)	0 (0 – 10)	0 (0 – 10)	0 (0 – 10)	0 (0 – 13)	0 (0 – 8)	0 (0 – 5)
Não	5 (0 – 23)	0 (0 – 25)	0 (0 – 24)	0 (0 – 12)	0 (0 – 15)	0 (0 – 10)	0 (0 – 22)	0 (0 – 10)	0 (0 – 13)	0 (0 – 17)
p-valor	0,002	<0,001	0,002	0,006	0,002	0,003	0,888	0,005	0,043	0,126
**Experiência de transfobia**										
Sim	7 (0 -29)	4 (0 - 25)	3 (0 – 30)	0 (0 -20)	0 (0 – 15)	0 (0 - 10)	0 (0 - 21)	0 (0 - 12)	0 (0 - 13)	0 (0 - 17)
Não	3 (0- 26)	0 (0 – 26)	0 (0 – 14)	0 (0 -20)	0 (0 – 10)	0 (0 - 10)	0 (0 - 22)	0 (0 - 13)	0 (0 - 7)	0 (0 - 3)
p-valor	0,099	0,121	0,125	0,879	0,545	0,708	0,794	0,571	0,929	0,850
**Índice de massa corporal**										
Baixo Peso	3 (0 - 20)	0 (0 -17)	0 (0 - 10)	-	0 (0 – 6)	-	0 (0 -17)	0 (0 -6)	-	-
Eutrofia	8 (0 – 24)	5 (0 – 25)	4 (0 - 30)	0 (0 - 12)	0 (0 -15)	0 (0 – 10)	0 (0 – 21)	0 (0 -12)	0 (0 – 13)	0 (0- 17)
Excesso de peso	6 (0 – 29)	3 (0 -26)	3 (0 - 23)	0 (0 – 20)	0 (0 – 10)	0 (0 – 10)	0 (0 – 22)	0 (0 -13)	0 (0 – 8)	0 (0 -5)
p-valor	0,280	0,213	0,502	0,107	0,316	0,208	0,963	0,506	0,594	0,466
**Insatisfação corporal**										
< Mediana	5,5 (0 -29)	0 (0 -25)	3 (0 - 24)	0 (0 – 12)	0 (0 -15)	0 (0 – 10)	0 (0 -22)	0 (0 – 10)	0 (0 – 13)	0 (0 - 17)
≥ Mediana	7 (0 -26)	0 (0 -26)	2 (0 – 30)	0 (0 – 20)	0 (0 – 10)	0 (0 – 10)	0 (0 -18)	0 (0 – 13)	0 (0 – 12)	0 (0 – 12)
p-valor	0,693	0,124	0,897	0,668	0,096	0,962	0,412	0,789	0,431	0,655

Variáveis*	Álcool	Tabaco	Maconha	Cocaína	Anfetaminas	Inalantes	Hipnóticos	Alucinógenos	Opióides	Outras
**Saúde do sono**										
Sem insônia	5 (0 – 25)	3 (0 – 25)	0 (0 – 24)	0 (0 – 5)	0 (0 – 5)	0 (0 – 6)	0 (0 – 22)	0 (0 -7)	0 (0 – 5)	0 (0 – 5)
Com insônia	8 (0 - 29)	4 (0 – 26)	5 (0 – 30)	0 (0 – 20)	0 (0 – 15)	0 (0 – 10)	0 (0 – 21)	0 (0 – 13)	0 (0 – 13)	0 (0 – 13)
p-valor	0,016	0,329	0,009	0,308	0,093	0,654	0,111	0,0326	0,233	0,897
**Depressão**										
Não	4 (0 – 20)	3 (0 – 25)	0 (0 – 16)	0 (0 - 3)	0 (0 – 7)	0 (0 – 6)	0 (0 – 3)	0 (0 – 13)	0 (0 – 2)	0 (0 – 3)
Sim	7,5 (0 – 29)	3,5 (0 -26)	3 (0 – 30)	0 (0 -20)	0 (0 – 15)	0 (0 – 10)	0 (0 – 22)	0 (0 – 12)	0 (0 – 17)	0 (0 – 17)
p-valor	0,017	0,404	0,100	0,065	0,119	0,363	0,006	0,726	0,081	0,321
**Qualidade de vida**										
< Mediana	6 (0 – 24)	2 (0 – 25)	0 (0 – 30)	0 (0 – 20)	0 (0 – 6)	0 (0 – 6)	0 (0 – 12)	0 (0 – 12)	0 (0 – 5)	0 (0 – 17)
≥ Mediana	7,5 (0 – 29)	4 (0 – 26)	3 (0 – 24)	0 (0 – 20)	0 (0 – 15)	0 (0 – 10)	0 (0 – 22)	0 (0 – 13)	0 (0 – 13)	0 (0 – 12)
p-valor	0,125	0,234	0,074	0,759	0,126	0,779	0,358	0,407	0,030	0,667

* Mediana (mínimo e máximo); ** Abreviações: EM: Ensino Médio; FC: Fundamental Completo; M: Mestrado; D: Doutorado. [a] Comparado às mulheres transgênero; [b] Comparado aos homens transgêneros.
