# Supplementary material for: Factors associated with alcohol and other drug use among transgender people: a cross-sectional study, Espírito Santo, 2022-2024
Source: Epidemiol Serv Saude. 2026 May 16;35:e20250205. doi: 10.1590/S2237-96222026v35e20250205.en (PMC13686421; doi:10.1590/S2237-96222026v35e20250205.en)
Supplement: Supplementary file 2 [file 2237-9622-ress-35-e20250205-table-supplementary.pdf]

Supplementary table 1. Score (median, minimum and maximum) for Consumption of alcohol, tobacco, marijuana, cocaine, amphetamines, inhalants, hypnotics, hallucinogens, opioids and other drugs according to thr sociodemographic and health variable categories of the total transgender population resident in the state of Espírito Santo based on the Alcohol, Smoking and Substance Involvement Screening Test (ASSIST), 2022-2024, n=103

Variables*	Alcohol	Tobacco	Marijuana	Cocaine	Amphetamines	Inhalants	Hypnotics	Hallucinogens	Opioids	Other
**Biological sex**										
Male	7 (0 – 24)	1.5 (0 – 24)	5 (0 – 14)	0 (0 – 20)	0 (0 – 2)	0 (0 – 6)	0 (0 – 17)	0 (0 – 5)	0 (0 – 13)	1.5 (0 – 24)
Female	7 (0 – 29)	3 (0 – 26)	2 (0 – 30)	0 (0 – 20)	0 (0 – 15)	0 (0 – 10)	0 (0 – 22)	0 (0 – 13)	0 (0 – 13)	3 (0 – 26)
p-value	0.981	0.395	0.360	0.922	0.387	0.951	0.736	0.510	0.107	0.598
**Gender identity**										
Transgender man	6.5 (0 – 29)	3 (0 – 26)	1 (0 – 30)	0 (0 – 20)	0 (0 – 10)	0 (0 – 10)	0 (0 – 22)	0 (0 – 13)	0 (0 – 7)	0 (0 – 5)
Transgender woman	6.0 (0 – 24)	3 (0 – 22)	5 (0 – 14)	0 (0 – 20)	0 (0 – 2)	0 (0 – 6)	0 (0 – 18)	0 (0 – 5)	0 (0 – 12)	0 (0 – 17)
Non-binary	7.5 (0 – 27)	5.5 (0 – 25)	2 (0 – 23)	3 (0 – 12)	0 (0 – 15)[a,b]	0 (0 – 10)[a,b]	0 (0 – 17)	0 (0 – 10)[a]	0 (0 – 13)	0 (0 – 3)
p-value	0.902	0.446	0.729	0.437	0.013	0.011	0.434	0.022	0.275	0.535
**Age (years)**										
<24	5.5 (0 – 24)	3 (0 – 25)	2.5 (0 – 30)	0 (0 – 22)	0 (0 – 7)	0 (0 – 3)	0 (0 – 22)	0 (0 – 13)	0 (0 – 3)	0 (0 – 3)
≥24	7 (0 – 29)	5 (0 – 26)	2 (0 – 27)	0 (0 – 22)	0 (0 – 15)	0 (0 – 10)	0 (0 – 18)	0 (0 – 12)	0 (0 – 13)	0 (0 – 17)
p-value	0.710	0.150	0.527	0.063	0.941	0.053	0.741	0.088	0.178	0.303
**Employment activity**										
Employed	8 (0 – 29)	6 (0 – 26)	2 (0 – 30)	0 (0 – 20)	0 (0 – 10)	0 (0 – 10)	0 (0 – 18)	0 (0 – 13)	0 (0 – 12)	0 (0 – 17)
Student and/or unemployed	5.5 (0 – 27)	3 (0 – 25)	3 (0 – 23)	0 (0 – 12)	0 (0 – 15)	0 (0 – 10)	0 (0 – 22)	0 (0 – 10)	0 (0 – 13)	0 (0 – 3)
p-value	0.113	0.059	0.986	0.581	0.579	0.521	0.112	0.661	0.579	0.555
**Place of residence**										
Greater Vitória	7 (0 – 27)	3 (0 – 26)	2 (0 – 30)	0 (0 – 20)	0 (0 – 15)	0 (0 – 10)	0 (0 – 22)	0 (0 – 13)	0 (0 – 13)	0 (0 – 17)
State interior	8 (0 – 29)	3 (0 – 25)	3 (0 – 15)	0 (0 – 7)	0 (0 – 5)	0 (0 – 5)	0 (0 – 7)	0 (0 – 5)	0 (0 – 5)	0 (0 – 5)
p-value	0.761	0.986	0.796	0.571	0.231	0.843	0.294	0.753	0.791	0.684

Variables*	Alcohol	Tobacco	Marijuana	Cocaine	Amphetamines	Inhalants	Hypnotics	Hallucinogens	Opioids	Other
**Marital status**										
Single	7 (0 – 27)	3 (0 – 26)	2 (0 – 30)	0 (0 – 20)	0 (0 – 15)	0 (0 – 10)	0 (0 – 22)	0 (0 – 13)	0 (0 – 13)	0 (0 – 12)
Stable union	6 (0 – 29)	8 (0 – 25)	2.5 (0 – 27)	0 (0 – 3)	0 (0 – 6)	0 (0 – 3)	0 (0 – 9)	0 (0 – 12)	0 (0 – 3)	0 (0 – 17)
p-value	0.840	0.264	0.751	0.830	0.577	0.197	0.233	0.888	0.504	0.668
**Race/skin color**										
White	5 (0 – 26)	2 (0 – 26)	0 (0 – 18)	0 (0 – 20)	0 (0 – 15)	0 (0 – 10)	0 (0 – 21)	0 (0 – 10)	0 (0 – 13)	0 (0 – 17)
Black or Brown	7 (0 – 29)	4 (0 – 25)	3.5 (0 – 30)	0 (0 – 20)	0 (0 – 7)	0 (0 – 6)	0 (0 – 22)	0 (0 – 13)	0 (0 – 12)	2 (0 – 26)
p-valor	0.421	0.989	0.188	0.420	0.502	0.953	0.909	0.618	0.896	0.027
**Schooling**										
HS and/or EE	6 (0 – 27)	3 (0 – 26)	2 (0 – 30)	0 (0 – 20)	0 (0 – 10)	0 (0 – 10)	0 (0 – 22)	0 (0 – 13)	0 (0 – 8)	0 (0 – 5)
HE and/or M/D**	8 (2 – 29)	4 (0 – 25)	5 (0 – 15)	0 (0 – 12)	0 (0 – 15)	0 (0 – 10)	0 (0 – 21)	0 (0 – 10)	0 (0 – 13)	0 (0 – 17)
p-value	0.373	0.812	0.590	0.777	0.295	0.729	0.227	0.949	0.602	0.468
**Number of people in household**										
Lives alone	9 (0 – 29)	4 (0 – 25)	3 (0 – 27)	0 (0 – 12)	0 (0 – 15)	0 (0 – 10)	0 (0 – 22)	0 (0 – 13)	0 (0 – 13)	0 (0 – 17)
>1 person(s)	5 (0 – 26)	2.5 (0 – 26)	1 (0 – 30)	0 (0 – 20)	0 (0 – 10)	0 (0 – 10)	0 (0 – 21)	0 (0 – 10)	0 (0 – 12)	0 (0 – 120)
p-value	0.039	0.123	0.095	0.637	0.027	0.156	0.729	0.030	0.055	0.099
**Income (minimum wage)**										
<1	7 (0 – 14)	5 (0 – 24)	4 (0 – 17)	0 (0 – 12)	0 (0 – 15)	0 (0 – 10)	0 (0 – 22)	0 (0 – 21)	0 (0 – 7)	0 (0 – 3)
1-2	8 (0 – 27)	5 (0 – 26)	2.5 (0 – 27)	0 (0 – 20)	0 (0 – 10)	0 (0 – 10)	0 (0 – 22)	0 (0 – 10)	0 (0 – 13)	0 (0 – 5)
>2	5 (0 – 29)	5 (0 – 25)	0 (0 – 14)	0 (0 – 20)	0 (0 – 3)	0 (0 – 6)	0 (0 – 17)	0 (0 – 13)	0 (0 – 8)	0 (0 – 17)
p-value	0.321	0.321	0.412	0.737	0.235	0.277	0.419	0.466	0.104	0.969
**Presence of comorbidities**										
Yes	6 (0 – 24)	0 (0 – 25)	0 (0 – 27)	0 (0 – 20)	0 (0 – 6)	0 (0 – 6)	0 (0 – 21)	0 (0 – 12)	0 (0 – 12)	0 (0 – 17)
No	7 (0 – 29)	4 (0 – 26)	3 (0 – 30)	0 (0 – 20)	0 (0 – 15)	0 (0 – 10)	0 (0 – 22)	0 (0 – 13)	0 (0 – 13)	0 (0 – 5)
p-value	0.613	0.295	0.497	0.164	0.069	0.557	0.081	0.337	0.954	0.034

Variables*	Alcohol	Tobacco	Marijuana	Cocaine	Amphetamines	Inhalants	Hypnotics	Hallucinogens	Opioids	Other
**Hormone therapy**										
Yes	6 (0 – 29)	0 (0 – 26)	3 (0 – 30)	0 (0 – 20)	0 (0 – 10)	0 (0 – 10)	0 (0 – 22)	0 (0 – 13)	0 (0 – 12)	0 (0 – 17)
No	8 (0 – 27)	4.5 (0 – 25)	2 (0 – 24)	0 (0 – 12)	0 (0 – 15)	0 (0 – 10)	0 (0 – 17)	0 (0 – 10)	0 (0 – 13)	0 (0 – 3)
p-value	0.201	0.195	0.748	0.761	0.922	0.613	0.870	0.367	0.310	0.867
**Gender-affirming surgical procedure**										
Yes	5 (0 – 26)	4 (0 – 26)	0 (0 – 10)	0 (0 – 20)	0 (0 – 10)	0 (0 – 10)	0 (0 – 18)	0 (0 – 10)	0 (0 – 12)	0 (0 – 17)
No	7 (0 – 29)	3 (0 – 25)	3 (0 – 30)	0 (0 – 20)	0 (0 – 15)	0 (0 – 10)	0 (0 – 22)	0 (0 – 13)	0 (0 – 13)	0 (0 – 5)
p-value	0.133	0.458	0.045	0.384	0.807	0.336	0.670	0.148	0.362	0.240
**Tobacco smoking**										
Yes	9.5 (0 – 29)	15 (0 – 26)	5.5 (0 – 30)	0 (0 – 20)	0 (0 – 10)	0 (0 – 10)	0 (0 – 10)	0 (0 – 13)	0 (0 – 8)	0 (0 – 5)
No	5 (0 – 23)	0 (0 – 25)	0 (0 – 24)	0 (0 – 12)	0 (0 – 15)	0 (0 – 10)	0 (0 – 22)	0 (0 – 10)	0 (0 – 13)	0 (0 – 17)
p-value	0.002	<0.001	0.002	0.006	0.002	0.003	0.888	0.005	0.043	0.126
**Experience of transphobia**										
Yes	7 (0 -29)	4 (0 - 25)	3 (0 – 30)	0 (0 -20)	0 (0 – 15)	0 (0 - 10)	0 (0 - 21)	0 (0 - 12)	0 (0 - 13)	0 (0 - 17)
No	3 (0- 26)	0 (0 – 26)	0 (0 – 14)	0 (0 -20)	0 (0 – 10)	0 (0 - 10)	0 (0 - 22)	0 (0 - 13)	0 (0 - 7)	0 (0 - 3)
p-value	0.099	0.121	0.125	0.879	0.545	0.708	0.794	0.571	0.929	0.850
**Body mass index**										
Low weight	3 (0 - 20)	0 (0 -17)	0 (0 - 10)	-	0 (0 – 6)	-	0 (0 -17)	0 (0 -6)	-	-
Normal weight	8 (0 – 24)	5 (0 – 25)	4 (0 - 30)	0 (0 – 12)	0 (0 -15)	0 (0 – 10)	0 (0 – 21)	0 (0 -12)	0 (0 – 13)	0 (0- 17)
Overweight	6 (0 – 29)	3 (0 -26)	3 (0 - 23)	0 (0 – 20)	0 (0 – 10)	0 (0 – 10)	0 (0 – 22)	0 (0 -13)	0 (0 – 8)	0 (0 -5)
p-value	0.280	0.213	0.502	0.107	0.316	0.208	0.963	0.506	0.594	0.466
**Body dissatisfaction**										
< Median	5.5 (0 -29)	0 (0 -25)	3 (0 - 24)	0 (0 – 12)	0 (0 -15)	0 (0 – 10)	0 (0 -22)	0 (0 – 10)	0 (0 – 13)	0 (0 - 17)
≥ Median	7 (0 -26)	0 (0 -26)	2 (0 – 30)	0 (0 – 20)	0 (0 – 10)	0 (0 – 10)	0 (0 -18)	0 (0 – 13)	0 (0 – 12)	0 (0 – 12)
p-value	0.693	0.124	0.897	0.668	0.096	0.962	0.412	0.789	0.431	0.655

Variables*	Alcohol	Tobacco	Marijuana	Cocaine	Amphetamines	Inhalants	Hypnotics	Hallucinogens	Opioids	Other
**Sleep health**										
Without insomnia	5 (0 – 25)	3 (0 – 25)	0 (0 – 24)	0 (0 – 5)	0 (0 – 5)	0 (0 – 6)	0 (0 – 22)	0 (0 -7)	0 (0 – 5)	0 (0 – 5)
Insomnia	8 (0 - 29)	4 (0 – 26)	5 (0 – 30)	0 (0 – 20)	0 (0 – 15)	0 (0 – 10)	0 (0 – 21)	0 (0 – 13)	0 (0 – 13)	0 (0 – 13)
p-value	0.016	0.329	0.009	0.308	0.093	0.654	0.111	0.0326	0.233	0.897
**Depression**										
No	4 (0 – 20)	3 (0 – 25)	0 (0 – 16)	0 (0 - 3)	0 (0 – 7)	0 (0 – 6)	0 (0 – 3)	0 (0 – 13)	0 (0 – 2)	0 (0 – 3)
Yes	7.5 (0 – 29)	3.5 (0 -26)	3 (0 – 30)	0 (0 -20)	0 (0 – 15)	0 (0 – 10)	0 (0 – 22)	0 (0 – 12)	0 (0 – 17)	0 (0 – 17)
p-valor	0.017	0.404	0.100	0.065	0.119	0.363	0.006	0.726	0.081	0.321
**Quality of life**										
< Median	6 (0 – 24)	2 (0 – 25)	0 (0 – 30)	0 (0 – 20)	0 (0 – 6)	0 (0 – 6)	0 (0 – 12)	0 (0 – 12)	0 (0 – 5)	0 (0 – 17)
≥ Median	7.5 (0 – 29)	4 (0 – 26)	3 (0 – 24)	0 (0 – 20)	0 (0 – 15)	0 (0 – 10)	0 (0 – 22)	0 (0 – 13)	0 (0 – 13)	0 (0 – 12)
p-value	0.125	0.234	0.074	0.759	0.126	0.779	0.358	0.407	0.030	0.667

* Median (minimum and maximum); ** Abbreviations: HS: High School; EE: Elementary Education; HE: High Education; M: Master's; D: Doctorate. [a] Compared to transgender women; [b] Compared to transgender men.
