# Supplementary material for: Factors associated with alcohol and other drug use among transgender people: a cross-sectional study, Espírito Santo, 2022-2024
Source: Epidemiol Serv Saude. 2026 May 16;35:e20250205. doi: 10.1590/S2237-96222026v35e20250205.en (PMC13686421; doi:10.1590/S2237-96222026v35e20250205.en)
Supplement: Translation

# Translation: Fatores associados ao consumo de álcool e de outras drogas entre pessoas transgênero: estudo transversal, Espírito Santo, 2022-2024

**DOI:** 10.1590/S2237-96222026v35e20250205.pt

**Authors:** Juliana Almeida Moreira, Beatriz Souza Silva, Weber Eugênio Birchler, Kaio Henrique Cesconetto Coswosck, Neide Aparecida Tosato Boldrini, Valdete Regina Guandalini, José Luiz Marques-Rocha

**Affiliations:** 1 Universidade Federal do Espírito Santo, Programa de Pós Graduação em Nutrição Saúde, Vitoria, ES, Brasil Universidade Federal do Espírito Santo Programa de Pós Graduação em Nutrição Saúde Vitoria ES Brasil; 2 Universidade Federal do Espírito Santo, Centro de Ciências da Saúde, Vitória, ES, Brasil Universidade Federal do Espírito Santo Centro de Ciências da Saúde Vitória ES Brasil

**Keywords:** Identidade de Gênero, Uso de Substâncias, Inequidade Social, Cirurgia de Afirmação de Gênero, Estudos Transversais

## Abstract

**Objetivo:**

Identificar os fatores associados ao consumo de álcool e de outras drogas em pessoas transgênero residentes no Espírito Santo.

**Métodos:**

Estudo transversal realizado com adultos transgênero a partir de questionário estruturado, onde foram coletadas variáveis sociodemográficas e de saúde, além da presença de sintomas depressivos, nível de qualidade de vida, saúde do sono e insatisfação corporal. O consumo de substâncias foi avaliado pela ferramenta teste de triagem para envolvimento com álcool, tabagismo e substâncias (ASSIST). Modelos de regressão linear multivariada ajustada por fatores de confusão foram analisados, sendo considerado o nível de significância de 5,0% para todos os testes.

**Resultados:**

A amostra final foi composta por 103 pessoas transgênero, com mediana de idade de 24 anos (18-44 anos). As drogas com maiores pontuações e maior necessidade de intervenção foram tabaco (45,6%), maconha (42,7%) e álcool (32,0%). Além disso, aproximadamente 10,0% da amostra apresentaram necessidade de intervenção para substâncias ilícitas. A gravidade dos sintomas depressivos, não realização da cirurgia de afirmação de gênero, renda, escolaridade, presença de comorbidades, idade, qualidade de vida e ser fumante associaram-se ao aumento do consumo de uma ou mais substâncias avaliadas (p-valor<0,050).

**Conclusão:**

Parte importante dos participantes fazia uso abusivo de uma ou mais das drogas avaliadas. Além disso, o consumo das substâncias avaliadas esteve associado a múltiplos determinantes sociais e de saúde, que atuam de forma interseccional, reforçando a vulnerabilidade dessa população frente ao uso de drogas.

Aspectos éticos

Esta pesquisa respeitou os princípios éticos, obtendo os seguintes dados de aprovação:

Comitê de ética em pesquisa:Universidade Federal do Espírito Santo

Número do parecer:4.431.995

Data de aprovação: 1/12/2020

Certificado de apresentação de apreciação ética:36320620.6.0000.5060

Registro do consentimento livre e esclarecido:Obtido de todos os participantes.

## Introdução 

O termo transgênero não é universal e nem consensual no Brasil. Por esse motivo, a palavra genérica "transgênero" comporta pessoas cuja identidade e/ou expressão de gênero é divergente das expectativas culturais com base no sexo biológico que lhes foi atribuído ao nascimento ^(^[xref ref-type="bibr" rid="r1"]^1^[/xref]^)^. Essas pessoas estão expostas a múltiplas vulnerabilidades sociais que impactam negativamente a saúde mental e sua qualidade de vida ^(^[xref ref-type="bibr" rid="r1"]^1^[/xref]^,^[xref ref-type="bibr" rid="r2"]^2^[/xref]^)^. Tais experiências negativas e recorrentes as colocam em risco substancial de fazerem uso abusivo de substâncias lícitas e ilícitas, de desenvolverem ansiedade, depressão e autolesão não suicida, além do risco de cometerem suicídio ^(^[xref ref-type="bibr" rid="r1"]^1^[/xref]^,^[xref ref-type="bibr" rid="r3"]^3^[/xref]^)^.

Apesar dos avanços das pesquisas nos últimos anos, observa-se predomínio de estudos que avaliaram o consumo de álcool e de tabaco por esse segmento populacional, sendo menos frequente a investigação sobre o uso de outras substâncias. Uma revisão de escopo sobre o uso de substâncias entre pessoas transgênero jovens demonstrou que 79,0% dos estudos se concentraram no uso do álcool, enquanto apenas 12,0% examinaram o uso de opioides ^(^[xref ref-type="bibr" rid="r4"]^4^[/xref]^)^.O histórico de experiências negativas de assistência à saúde, como discriminação dos profissionais, barreiras no acesso às ações e aos serviços de saúde e de cuidado, bem como a dificuldade do reconhecimento legal ao gênero, constituem fatores que podem culminar na utilização de substâncias lícitas e ilícitas como mecanismo de enfrentamento ^(^[xref ref-type="bibr" rid="r4"]^4^[/xref]^)^. Embora limitados, os estudos existentes indicam diferenças relevantes no uso de drogas entre pessoas transgênero, quando comparado à população cisgênero ^(^[xref ref-type="bibr" rid="r5"]^5^[/xref]^,^[xref ref-type="bibr" rid="r6"]^6^[/xref]^)^

As barreiras no acesso aos serviços de saúde e a discriminação contribuem para a automedicação e dificultam o cuidado adequado desse público ^(^[xref ref-type="bibr" rid="r7"]^7^[/xref]^)^. Em contextos específicos, como o trabalho sexual e o uso não supervisionado de hormônios, o risco de consumo problemático de drogas pode ser ainda maior. Ressalta-se que o Brasil tem um dos maiores números de homicídios de pessoas transgênero do mundo ^(^[xref ref-type="bibr" rid="r8"]^8^[/xref]^)^ e, além da violência explícita, elas ainda encontram barreiras para acessar os serviços públicos básicos ^(^[xref ref-type="bibr" rid="r7"]^7^[/xref]^)^.

Nesse sentido, faz-se necessário um estudo mais aprofundado das características únicas e das vivências desse segmento populacional para identificação dos fatores associados ao consumo de substâncias lícitas e ilícitas. Nesse contexto, investigar o consumo de drogas entre pessoas transgênero é fundamental para ampliar a compreensão sobre suas demandas de saúde e subsidiar intervenções baseadas na equidade, na redução de danos e na promoção dos direitos humanos. Portanto, este estudo tem por objetivo identificar os fatores associados ao consumo de álcool e de outras drogas em pessoas transgênero.

## Métodos

Desenho do estudo

Trata-se de estudo transversal descritivo, envolvendo indivíduos transgênero residentes no estado do Espírito Santo.

População e amostra

O convite para participar do estudo se deu por meio de link eletrônico da plataforma Google Forms, divulgado através de mídias digitais (Instagram, Facebook, WhatsApp e e-mail) em associações voltadas ao segmento LGBTQIAPN+ e para os participantes do Ambulatório de Diversidade de Gênero do Hospital Universitário Cassiano Antônio Moraes. Foram incluídos no estudo aqueles que selecionaram a opção "homem transgênero ou mulher transgênero ou gênero não binário" como sua identidade de gênero. Foram excluídos das análises os menores de 18 anos e os não residentes no Espírito Santo. A amostra final, calculada pelo software G-Power, foi representativa da população transgênero do estado com intervalo de confiança de 95% (IC95%) e erro amostral de 9,5% ^(^[xref ref-type="bibr" rid="r9"]^9^[/xref]^)^. Esses cálculos foram realizados a partir da prevalência de pessoas transgênero/não binárias no Brasil (2,0%) ^(^[xref ref-type="bibr" rid="r10"]^10^[/xref]^)^ e do número de habitantes no estado ^(^[xref ref-type="bibr" rid="r11"]^11^[/xref]^)^.

Coleta de dados

Os voluntários responderam aos questionários no período de julho de 2022 a julho de 2024 de forma anônima, visando a uma maior honestidade nas respostas, reduzindo o viés de respostas socialmente desejáveis. Os participantes que tiveram qualquer diagnóstico ou indicativo de alguma comorbidade ou condição de sofrimento foram encaminhados para a unidade de saúde de referência, para que fossem atendidos por equipe multiprofissional.

Variáveis sociodemográficas

A idade foi determinada em anos e posteriormente categorizada segundo a mediana (<24 e ≥24 anos). A raça/cor da pele foi autodeclarada segundo o Instituto Brasileiro de Geografia e Estatística ^(^[xref ref-type="bibr" rid="r12"]^12^[/xref]^)^ e categorizada em "branca" e "preta e/ou parda". O sexo atribuído ao nascimento foi categorizado como "masculino" ou "feminino". A escolaridade foi categorizada em "ensino fundamental completo", "ensino médio completo" ou "ensino superior completo/mestrado/doutorado". O estado civil foi classificado com "união estável" ou "solteiro(a)". A ocupação foi categorizada como "estudante", "empregado" ou "desempregado". A renda familiar (per capita) foi categorizada em <1 salário mínimo, 1 a 2 salários mínimos e >2 salários mínimos, de acordo com os valores vigentes nos respectivos anos. O local de moradia foi categorizado como "interior" ou "capital" ^(^[xref ref-type="bibr" rid="r13"]^13^[/xref]^)^.

Variáveis de saúde

A presença de comorbidades foi autorreferida e categorizada em "sim" ou "não". O histórico de cirurgia foi avaliado com o questionamento de que já se havia realizado alguma cirurgia para afirmação de gênero. A variável acompanhamento médico foi categorizada em "sim" ou "não". O acompanhamento com outro profissional de saúde foi determinado caso fosse feito acompanhamento com nutricionista, fonoaudiólogo, psicólogo/psiquiatra ou acompanhamento com mais de um profissional.

A massa corporal foi autorreferida em quilos e a estatura em metros. Dessa forma, o estado nutricional foi determinado por meio do índice de massa corporal (IMC), segundo os critérios da Organização Mundial da Saúde (OMS) ^(^[xref ref-type="bibr" rid="r14"]^14^[/xref]^)^ e categorizado em "baixo peso", "eutrofia" ou "excesso de peso".

A experiência de transfobia foi determinada de forma autorreferida e categorizada em "sim" ou "não". A hormonização foi indicada de forma autorreferida em "sim" ou "não", e o tempo de hormonização foi classificado em <2 anos e ≥2 anos.

A percepção da autoimagem foi avaliada por meio das Escalas de Silhuetas Brasileiras para Adultos e Crianças ^(^[xref ref-type="bibr" rid="r15"]^15^[/xref]^)^. A autopercepção de sentimentos e de depressão foi obtida pelo inventário de Beck ^(^[xref ref-type="bibr" rid="r16"]^16^[/xref]^)^, instrumento que consiste em 21 grupos de afirmações e é graduado de 0 a 3 pontos, com afirmações sobre como o participante está se sentindo na última semana. Os resultados são expressos em pontos e classificados em quatro grupos: (a) 0-11, sem depressão; (b) 12-19, depressão leve; (c) 20-35 depressão moderada; (d) 36-63 depressão grave. Posteriormente, esses grupos foram categorizados em "sem depressão (pontuação 0-11)" e "presença de depressão (pontuação ≥12)".

A qualidade de vida foi avaliada pelo questionário Short-Form 6 dimensions (SF-6D) para descrever estados de saúde avaliando-se seis dimensões: capacidade funcional; aspectos sociais; aspectos físicos e emocionais; dor; saúde mental; vitalidade. As pontuações variam de 0 a 1, em que 0 representa um pior estado de saúde e 1 indica um melhor estado de saúde ^(^[xref ref-type="bibr" rid="r17"]^17^[/xref]^)^, o que é depois categorizado segundo a mediana. Por fim, a qualidade do sono também foi avaliada, a partir da escala PROMIS ^(^[xref ref-type="bibr" rid="r18"]^18^[/xref]^)^, uma versão adaptada e validada para medir distúrbios do sono, sendo dicotomizada em "sem insônia" (pontuação <25) ou "insônia" (pontuação ≥25).

Variável desfecho

A variável consumo de álcool e de outras drogas foi obtida a partir da aplicação do teste de triagem para envolvimento com álcool, tabagismo e substâncias (ASSIST) ^(^[xref ref-type="bibr" rid="r19"]^19^[/xref]^)^, devido à inexistência de escalas validadas para a população transgênero. O ASSIST foi desenvolvido pela OMS e validado para a população brasileira como um questionário de triagem composto por oito questões. Aqueles com a pontuação ≤10 para álcool e ≤3 para outras drogas foram categorizados em "sem intervenção". Por outro lado, foram categorizados em "receber intervenção" quando a pontuação foi ≥4 para drogas e ≥11 para álcool ^(^[xref ref-type="bibr" rid="r19"]^19^[/xref]^)^.

Análise de dados

A normalidade dos dados foi verificada pelo teste de Shapiro-Wilk. Para caracterização da amostra, as variáveis contínuas foram expressas por meio da média, do desvio padrão e da mediana (mínimo-máximo), e as variáveis categóricas em percentuais. Foi realizada a análise de dados ausentes, por meio do método expectation-maximization, para evitar viés decorrente de exclusões por dados incompletos.

As diferenças entre as categorias de identidade de gênero foram exploradas usando os testes qui-quadrado e exato de Fisher, para diminuir os riscos de erros do tipo I ou II. Para a avaliação da pontuação de álcool e de outras drogas segundo as variáveis sociodemográfica e de saúde, foi realizada análise variância unidirecional e teste de Kruskal-Wallis. A análise de regressão linear multivariada foi aplicada para determinar a influência das variáveis independentes no consumo de álcool e de outras drogas. As variáveis de natureza sociodemográfica e de saúde utilizadas na regressão linear foram incluídas por serem potenciais confundidoras. Essa abordagem permitiu ajuste dos efeitos das variáveis independentes, controlando o impacto de fatores que poderiam distorcer a associação entre identidade de gênero e consumo de substâncias lícitas e ilícitas. Todas as variáveis que apresentaram p-valor<0,200 nas análises bivariadas foram incluídas. Modelos brutos e ajustados para idade, raça/cor da pele, escolaridade, renda, identidade de gênero e estado civil foram analisados. As análises estatísticas foram conduzidas pelo SPSS v.22, com o nível de significância de 5,0%.

## Resultados 

A amostra final contou com 103 indivíduos transgênero, sendo 64 homens transgênero (62,1%), 21 mulheres transgênero (20,3%) e 18 não binários (17,4%) (Tabela 1). A idade mediana dos participantes foi de 24 anos (18-44 anos), não apresentando diferença entre as identidades de gênero. Houve maior proporção de pessoas de raça/cor da pele preta ou parda (60,2%), estudantes e/ou desempregados(as) (52,4%), residindo na capital do estado (79,6%), solteiros(as) (79,2%) e com ensino médio e/ou fundamental (80,6%). Além disso, 44,9% dos indivíduos possuíam renda *per capita* entre 1 e 2 salários mínimos, 55,3% estavam em terapia hormonal para afirmação de gênero, sendo que, destes, apenas 32,3% estavam em hormonioterapia por um período igual ou superior a dois anos, e 35,9% utilizavam hormônios sem prescrição médica. Quanto ao estado nutricional, aproximadamente metade da amostra (49,5%) foi classificada com excesso de peso segundo o IMC.

As pontuações para as substâncias lícitas e ilícitas avaliadas indicaram que as drogas com maior necessidade de intervenção foram tabaco (45,6%), maconha (42,7%) e álcool (32,0%) (Tabela 2). Além disso, aproximadamente 10% da amostra apresentarou necessidade de intervenção para o uso de cocaína, anfetaminas, alucinógenos e hipnóticos. Quando as pontuações foram comparadas entre as identidades de gênero, verificou-se um consumo mais elevado de anfetaminas e inalantes entre pessoas não binárias, quando comparadas às demais categorias (p-valor 0,013). O mesmo ocorreu para o consumo de alucinógenos, que foi superior entre não binários em comparação às mulheres transgênero (p-valor 0,022). Para as demais substâncias, não foram observadas diferenças estatisticamente significativas.

No intuito de triar as variáveis sociodemográficas e de saúde da população transgênero associadas ao consumo de álcool e de outras drogas avaliadas, foi elaborada uma tabela (Tabela Suplementar 1) que apresenta as pontuações para as substâncias de acordo com as categorias dessas variáveis. Os resultados revelaram um grande número de p-valor<0,200, demonstrando a relação entre o consumo das substâncias e as variáveis estudadas. A partir desses dados, realizaram-se as análises de regressão linear multivariada.

As análises de regressão linear multivariada ajustada por possíveis fatores de confusão indicaram que o consumo de substâncias lícitas e ilícitas está associado a: sintomas depressivos, nível de qualidade de vida, tabagismo, escolaridade, renda, comorbidades, realização de cirurgia de adequação de gênero, sexo designado ao nascimento, idade, número de pessoas residentes e ocupação (p-valor<0,050 para todos) (Tabela 3).

Verificou-se, no modelo ajustado, que a qualidade de vida (β 16,056; IC95% 2,946; 29,166), o hábito tabagista (β 3,969; IC95% 6,199; 20,027), ser estudante e/ou estar desempregado(a) (β 3,773; IC95% 1,181; 6,365), além da pontuação de sintomas depressivos (β 0,186; IC95% 0,073; 0,299), se associaram ao aumento do consumo de álcool (Tabela 3). Da mesma forma, ser fumante (β 3,832; IC95% 1,217; 6,446), não ter realizado cirurgia de adequação de gênero (β 2,041; IC95% 0,642; 3,440) e a pontuação dos sintomas depressivos (β 0,114; IC95% 0,007; 0,220) estiveram associados ao aumento no consumo de maconha pelos participantes do estudo.

O tabagismo teve uma associação com a idade de forma isolada, mesmo após os demais ajustes, em que o aumento da idade se relacionou com as maiores pontuações para o tabaco (β 0,352; IC95% 0,072; 0,632). O hábito de fumar esteve ainda associado ao maior consumo de cocaína (β 2,041; IC95% 0,642; 3,440), alucinógenos (β 1,707; IC95% 0,246; 2,206) e anfetaminas (β 1,022; IC95% 0,088; 1,957). Para essa última substância, a renda inferior a um salário mínimo mostrou associação negativa (β -0,590; IC95% -1,268; -0,050) (Tabela 3).

O sexo indicado no nascimento se associou de forma diferente, a depender da substância avaliada. Ser designado(a) pelo sexo biológico feminino no nascimento associou-se positivamente ao consumo de inalantes (β 0,572; IC95% 0,106; 1,039) e de forma negativa ao uso de opioides (β -1,211; IC95% -2,240; -0,181).

Quanto ao consumo de hipnóticos, verificou-se que a pontuação para sintomas depressivos esteve positivamente associada (β 0,086; IC95% 0,014; 0,158), enquanto ser estudante e desempregado(a) apresentou associação inversa (β -1,707; IC95% -3,340; -0,075). Do mesmo modo, morar sozinho(a) esteve negativamente relacionado ao consumo de alucinógenos (β -1,231; IC95% -2,206; -0,265). Adicionalmente, ter realizado a cirurgia de afirmação de gênero (β -1,746; IC95% -2,841; -0,651) ou apresentar comorbidades (β -1,192; IC95% -2,080; -0,304) associaram-se de forma inversa com o consumo de outras substâncias, enquanto aqueles com menor grau de escolaridade (β 1,255; IC95% 0,294; 2,217) apresentaram associação positiva ao consumo de outras substâncias (Tabela 3).

**Tabela 1:** Características dos participantes no total e segundo identidade de gênero. Espírito Santo, 2022-2024 (n=103)

Variáveis	Total n (%)	Mulher transgênero n (%)	Homem transgênero n (%)	Não binário n (%)	p-valor
Total	103 (100,0)	21 (20,3)	64 (62,1)	18 (17,4)	
Idade					0,958
<24 anos	44 (42,7)	10 (45,5)	27 (41,5)	8 (43,8)	
≥24 anos	59 (57,3)	11 (54,5)	37 (58,5)	10 (56,3)	
Raça/cor da pele					0,360
Branco(a)	41 (39,8)	6 (28,6)	29 (45,3)	6 (33,3)	
Preto e/ou pardo(a)	62 (60,2)	15 (71,4)	35 (54,7)	12(66,7)	
Ocupação					0,057
Estudante e/ou desempregado(a)	54 (52,4)	9 (42,9)	31 (48,4)	14(77,8)	
Empregado(a)	49 (47,6)	12 (57,1)	33 (51,6)	4 (22,2)	
Escolaridade					0,047
Ensino médio ou fundamental completo	83 (80,6)	13 (61,9)	55 (85,9)	15 (83,3)	
Ensino superior/mestrado/doutorado	20 (19,4)	8 (38,1)	9 (14,1)	3 (16,7)	
Renda (salários mínimos)a					0,001
<1	17 (17,3)	3 (15,0)	7 (11,3)	7 (43,8)	
1-2	44 (44,9)	4 (20,0)	35 (56,5)	5 (31,3)	
>2	37 (37,8)	13 (65,0)	20 (32,3)	4 (25,0)	
Estado civil					0,081
Solteiro(a)	81 (79,2)	19 (90,5)	45 (70,4)	17 (94,4)	
União estável	22 (21,4)	2 (9,5)	19 (29,7)	1 (5,6)	
Cidade					0,508
Capital	82 (79,6)	15 (71,4)	53 (82,8)	14 (77,8)	
Interior	21 (20,4)	6 (28,6)	11 (17,2)	4 (22,2)	
Hormonioterapia					0,004
Sim	57 (55,3)	15 (71,4)	38 (59,4)	4 (22,2)	
Não	46 (44,7)	6 (28,6)	26 (40,6)	14 (77,8)	
Hormonioterapia sem prescrição					<0,001
Sim	37 (35,9)	15 (71,4)	19 (29,7)	3 (16,7)	
Não	66 (64,1)	6 (28,6)	45 (70,3)	15 (83,3)	
Tempo de hormonização (anos)					0,028
<2	21 (21,2)	5 (25)	13 (21,3)	3 (16,7)	
≥2	32 (32,3)	9 (45,0)	22 (36,1)	1 (5,6)	
Não faz	46 (46,5)	6 (30,0)	26 (42,6)	14 (77,8)	
Cirurgia de afirmação de gênerob					0,523
Sim	15 (14,7)	4 (19,0)	10 (15,6)	1 (5,9)	
Não	87 (85,3)	17 (81,0)	54 (84,4	16 (94,1)	
Experiência de transfobia					0,069
Sim	91 (88,3)	21 (100,0)	53 (82,8)	17 (94,4)	
Não	12 (11,7)	0 (0,0)	11 (17,2)	1 (5,6)	
Índice de massa corporal					0,106
Baixo peso	9 (8,7)	3 (14,3)	3 (4,7)	3 (16,7)	
Eutrofia	43 (41,7)	12 (57,1)	24 (37,5)	7 (38,9)	
Excesso de peso	51 (49,5)	6 (28,6)	37 (57,8)	8 (44,4)	
Presença de comorbidade(s)					0,946
Sim	25 (24,3)	5 (23,8)	15 (23,4)	5 (27,8)	
Não	78 (75,7)	16 (76,2)	49 (76,6)	13 (72,2)	
Depressão					0,523
Sim	75 (73,0)	17 (79,2)	44 (69,2)	14 (80)	
Não	28 (27,0)	4 (20,8)	20 (30,8)	4 (20)	
Saúde do sono					0,615
Ausência de insônia	35 (34,0)	9 (40,9)	20 (30,8)	7 (37,5)	
Presença de insônia	68 (76,0)	12 (59,1)	44 (69,2)	11 (62,5)	
Qualidade de vida					0,350
Abaixo da mediana	51 (49,5)	9 (36,4)	34 (53,8)	8 (43,8)	
Acima ou igual a mediana	52 (50,5)	16 (64,6)	30 (46,2)	10 (56,2)	

a n=98; ^b^n=101.

**Tabela 2:** Pontuação (mediana: mínimo e máximo) e classificação da necessidade de intervenção (%) para consumo de álcool e outras drogas na população total e por identidade de gênero. Espírito Santo, 2022-2024 (n=103)

Variáveis	Total n (%)	Mulher transgênero n (%)	Homem transgênero n (%)	Não binário n (%)	p-valor
Álcool (pontuação)	7 (0;29)	6 (0;24)	6,5 (0;29)	7,5 (0;27)	0,902
Álcool (classificação)					0,473
Sem intervenção	70 (68,0)	15 (71,4)	45 (70,3)	10 (55,6)	
Receber intervenção	33 (32)	6 (28,6)	19 (29,7)	8 (44,4)	
Tabaco (pontuação)	3 (0;26)	3 (0;22)	3 (0;26)	5,5 (0;25)	0,446
Tabaco (classificação)					0,586
Sem intervenção	56 (54,4)	13 (61,9)	35 (54,7)	8 (44,4)	
Receber intervenção	47 (45,6)	8 (38,1)	29 (45,3)	10 (55,6)	
Maconha (pontuação)	2 (0;30)	5 (0;14)	1 (0;30)	2 (0;23)	0,729
Maconha (classificação)					0,341
Sem intervenção	59 (57,3)	9 (42,9)	39 (60,9)	11 (61,1)	
Receber intervenção	44 (42,7)	12 (57,1)	25 (39,1)	7 (38,9)	
Cocaína (pontuação)	0 (0;20)	0 (0;20)	0 (0;20)	0 (0;12)	0,437
Cocaína (classificação)					0,424
Sem intervenção	94 (91,3)	19 (90,5)	60 (93,8)	15 (83,3)	
Receber intervenção	9 (8,7)	2 (9,5)	4 (6,3)	3 (16,7)	
Anfetaminas (pontuação)	0 (0;15)	0 (0;2)	0 (0;10)	0 (0;15)a,b	0,013
Anfetaminas (classificação)					0,126
Sem intervenção	96 (93,2)	21 (100)	60 (93,8)	15 (83,3)	
Receber intervenção	7 (6,8)	0 (0)	4 (6,3)	3 (16,7)	
Inalantes (pontuação)	0 (0;10)	0 (0;6)	0 (0;9)	0 (0;10)a,b	0,011
Inalantes (classificação)					0,350
Sem intervenção	98 (95,1)	20 (95,2)	62 (96,9)	16 (88,9)	
Receber intervenção	5 (4,9)	1 (4,8)	2 (3,1)	2 (11,1)	
Hipnóticos (pontuação)	0 (0;22)	0 (0;18)	0 (0;22)	0 (0;17)	0,434
Hipnóticos (classificação)					0,554
Sem intervenção	89 (86,4)	19 (90,5)	56 (87,5)	14 (76,8)	
Receber intervenção	14 (13,6)	2 (9,5)	8 (12,5)	4 (22,2)	
Alucinógenos (pontuação)	0 (0;13)	0 (0;5)	0 (0;13)	0 (0;10)a	0,022
Alucinógenos (classificação)					0,476
Sem intervenção	94 (91,3)	20 (95,2)	59 (92,2)	15 (83,3)	
Receber intervenção	9 (8,7)	1 (4,9)	5 (7,8)	3 (16,7)	
Opioides (pontuação)	0 (0;13)	0 (0;12)	0 (0;7)	0 (0;13)	0,275
Opioides (classificação)					0,559
Sem intervenção	99 (96,1)	20 (95,2)	62 (96,9)	17 (94,4)	
Receber intervenção	4 (3,9)	1 (4,8)	2 (3,1)	1 (5,6)	
Outrasdrogas (pontuação)	0 (0;17)	0 (0;17)	0 (0;5)	0 (0;3)	0,535
Outras drogas (classificação)					0,183
Sem intervenção	100 (97,1)	19 (90,5)	63 (98,4)	18 (100)	
Receber intervenção	3 (2,9)	2 (9,5)	1 (1,6)	(0)	

a Comparado às mulheres transgênero; ^b^ Comparado aos homens transgêneros.

**Tabela 3:** Coeficiente (β) e intervalos de confiança de 95% (IC95%) brutos e ajustados entre a pontuação do consumo de álcool e outras drogas e variáveis independentes. Espírito Santo, 2022-2024 (n=103)

Variáveis	β bruto (IC95%)	p-valor	β ajustado (IC95%)	p-valor
Álcool				
Qualidade de vida	16,786 (3,968; 29,604)	0,011	16,056 (2,946; 29,166)	0,017
Tabagismo	4,335 (1,666; 7,003)	0,002	3,969 (6,199; 20,027)	0,004
Estudante e/ou desempregado	3,612 (1,085; 6,139)	0,006	3,773 (1,181; 6,365)	0,005
Pontuação de sintomas depressivos	0,179 (0,069; 0,289)	0,002	0,186 (0,073; 0,299)	0,002
Tabaco				
Idade	0,337 (0,057; 0,616)	0,019	0,352 (0,072; 0,632)	0,014
Maconha				
Tabagismo	4,715 (2,084; 7,346)	0,001	3,832 (1,217; 6,446)	0,005
Não fez cirurgia de adequação de gênero	3,438 (0,003; 6,874)	0,050	2,041 (0,642; 3,440)	0,040
Pontuação de sintomas depressivos	0,115 (0,008; 0,223)	0,036	0,114 (0,007; 0,220)	0,037
Cocaína				
Ser fumante	1,990 (0,649; 3,331)	0,004	2,041 (0,642; 3,440)	0,005
Anfetaminas				
Ser fumante	1,022 (0,088; 1,957)	0,032	1,022 (0,088; 1,957)	0,032
<1 salário mínimo	-0,659 (-1,268; 0,050)	0,034	-0,590 (-1,260; 0,051)	0,034
Inalantes				
Sexo biológico feminino	0,572 (0,106; 1,039)	0,017	0,572 (0,106; 1,039)	0,017
Hipinóticos				
Pontuação de sintomas depressivos	0,086 (0,014; 0,158)	0,020	0,086 (0,014; 0,158)	0,020
Estudante e/ou desempregado	-1,707 (-3,340; -0,075)	0,041	-1,707 (-3,340; -0,075)	0,041
Alucinógenos				
Ser fumante	1,716 (0,725; 2,706)	0,001	1,707 (0,246; 2,206)	0,002
Morar sozinho	-1,224 (-2,157; 0,291)	0,011	-1,231 (-2,206; -0,265)	0,014
Opioides				
Sexo biológico feminino	-1,496 (-2,563; -0,430)	0,006	-1,211 (-2,240; -0,181)	0,022
Outras substâncias				
Fez cirurgia de adequação de gênero	-1,513 (-2,622; -0,403)	0,008	-1,746 (-2,841; -0,651)	0,002
Presença de comorbidades	-1,209 (-2,121; -0,297)	0,010	-1,192 (-2,080; -0,304)	0,009
Ensino fundamental completo/ensino médio completo	1,211 (0,223; 2,198)	0,017	1,255 (0,294; 2,217)	0,011

## Discussão

Os resultados deste estudo evidenciaram que a gravidade dos sintomas depressivos, a não realização da cirurgia de afirmação de gênero, a renda, a escolaridade, a presença de comorbidades, a idade, a qualidade de vida e ser fumante associaram-se ao aumento do consumo de uma ou mais substâncias lícitas e ilícitas avaliadas. Dentre elas, as mais frequentemente consumidas foram tabaco, maconha e álcool. Adicionalmente, todas as demais substâncias investigadas apresentaram pontuações indicativas de necessidade de intervenção, evidenciando um padrão de uso potencialmente abusivo de substâncias. Esses resultados corroboram os dados de uma pesquisa do tipo coorte longitudinal que acompanhou jovens de minorias sexuais e de gênero norte-americanos designados como homens no nascimento ^(^[xref ref-type="bibr" rid="r20"]^20^[/xref]^)^. Nesse estudo, aproximadamente 4,7% dos participantes apresentaram alto risco de transtorno por uso de álcool, 14,0% apresentaram pontuações compatíveis com possível transtorno por uso de cannabis, 9,8% relataram uso de estimulantes nos últimos seis meses, 7,0% relataram uso de drogas em casas noturnas e 10,7% relataram uso de quaisquer outras drogas ilícitas ^(^[xref ref-type="bibr" rid="r20"]^20^[/xref]^)^.

Uma revisão sistemática envolvendo 25.233 indivíduos transgênero adultos demonstrou que o estigma e a violência contra essas pessoas são comuns, e que o risco de sofrer esse tipo de discriminação está bem estabelecido e tem sido associado ao envolvimento em comportamentos de risco à saúde, como o uso de substâncias lícitas e ilícitas como meio de enfrentamento ^(^[xref ref-type="bibr" rid="r5"]^5^[/xref]^)^. O conceito de estresse minoritário sugere que membros de grupos sociais marginalizados vivenciam sobrecarga de estresse, o que pode contribuir para comportamentos de enfrentamento desadaptativos, como o uso de substâncias psicoativas ^(^[xref ref-type="bibr" rid="r21"]^21^[/xref]^)^. Nesse contexto, um estudo holandês envolvendo jovens transgênero demonstrou que altas taxas de uso de drogas entre minorias de gênero são frequentemente explicadas por suas experiências com estressores minoritários ^(^[xref ref-type="bibr" rid="r22"]^22^[/xref]^)^. Esse modelo argumenta que minorias sociais experimentam estressores específicos além dos estressores cotidianos ^(^[xref ref-type="bibr" rid="r23"]^23^[/xref]^)^.

Outro importante estudo, envolvendo adultos transgênero de Massachusetts e de Rhode Island, revelou que 60,5% dos participantes tiveram pelo menos uma experiência de abuso físico ou sexual na infância, 87,4% sofreram discriminação atribuída à identidade de gênero, enquanto 30,5% relataram histórico de violência por parceiro íntimo ^(^[xref ref-type="bibr" rid="r24"]^24^[/xref]^)^. As análises ainda revelaram que as altas pontuações de discriminação às pessoas transgênero foram positivamente associadas ao uso de substâncias ^(^[xref ref-type="bibr" rid="r24"]^24^[/xref]^)^. Os resultados do presente estudo corroboram os achados dos artigos supracitados, uma vez que muitas variáveis socioeconômicas e de saúde se associaram ao consumo de álcool e de outras drogas.

No Brasil, poucos estudos abordaram o consumo de álcool e de outras drogas por essa população. Em um desses estudos, realizado nas cinco regiões brasileiras, verificou-se que maconha, sedativos e tranquilizantes, assim como cocaína, anti-histamínicos e narguilé foram as substâncias mais usadas entre indivíduos transgênero ^(^[xref ref-type="bibr" rid="r25"]^25^[/xref]^)^. Segundo os autores, os principais fatores associados ao uso de substâncias foram a discriminação e a instabilidade no ambiente familiar. Em outro estudo, realizado com mulheres transgênero brasileiras, foi observada uma maior propensão ao consumo de drogas ilícitas antes ou durante o sexo, com o objetivo de potencializar, desinibir ou facilitar a experiência sexual ^(^[xref ref-type="bibr" rid="r26"]^26^[/xref]^)^. Essas relações demonstram a interseccionalidade de vulnerabilidades específicas desse segmento populacional. A amplitude do olhar investigativo se faz necessária, uma vez que a interseccionalidade visa apreender a complexidade das identidades e das desigualdades sociais por meio da integração com os principais eixos de diferenciação social, como sexo/gênero, classe, raça/cor da pele, etnia, idade, deficiência, orientação sexual e outros ^(^[xref ref-type="bibr" rid="r27"]^27^[/xref]^)^.

Estudos anteriores observaram que o álcool é uma das substâncias com maior presença entre a população ^(^[xref ref-type="bibr" rid="r28"]^28^[/xref]^,^[xref ref-type="bibr" rid="r29"]^29^[/xref]^)^, sendo que, em 2020, 60,4% dessa população relatou consumi-lo regularmente ^(^[xref ref-type="bibr" rid="r30"]^30^[/xref]^)^. O estigma que esse segmento populacional enfrenta e os eventos de preconceito leva a um maior risco de uso problemático de álcool ^(^[xref ref-type="bibr" rid="r31"]^31^[/xref]^)^. Em consonância com os estudos prévios, neste estudo, identificou-se uma associação linear entre a gravidade dos sintomas depressivos e o aumento do consumo de álcool, maconha e hipnóticos. O consumo de substâncias lícitas e ilícitas está intimamente relacionado ao surgimento ou ao agravamento de sintomas depressivos, em virtude de seus efeitos neurobiológicos e psicossociais ^(^[xref ref-type="bibr" rid="r32"]^32^[/xref]^)^. Do ponto de vista fisiopatológico, substâncias como o álcool atuam modulando neurotransmissores, inicialmente produzindo sensação de euforia ou de relaxamento e, posteriormente, com o uso repetido ou excessivo, gerando alterações na plasticidade neuronal e disfunções nos circuitos de recompensa e de humor ^(^[xref ref-type="bibr" rid="r32"]^32^[/xref]^,^[xref ref-type="bibr" rid="r33"]^33^[/xref]^)^. Tais alterações podem precipitar ou intensificar quadros depressivos, sobretudo em indivíduos expostos a estressores psicossociais ^(^[xref ref-type="bibr" rid="r34"]^34^[/xref]^)^. Além disso, o uso de drogas, frequentemente, surge como estratégia de alívio temporário para sintomas depressivos, criando um ciclo bidirecional em que o consumo mantém e agrava a sintomatologia depressiva, dificultando a recuperação clínica e aumentando o risco de complicações psiquiátricas e sociais ^(^[xref ref-type="bibr" rid="r32"]^32^[/xref]^,^[xref ref-type="bibr" rid="r34"]^34^[/xref]^)^.

Outro resultado relevante do presente estudo foi a associação positiva entre a não realização da cirurgia de afirmação de gênero e o consumo de maconha e de outras drogas. Um estudo norte-americano com 27.715 pessoas transgênero demonstrou que a realização de uma cirurgia de afirmação de gênero esteve associada à redução da probabilidade de sofrimento psicológico e do tabagismo ^(^[xref ref-type="bibr" rid="r35"]^35^[/xref]^)^. Porém, após ajustes, esse mesmo estudo não encontrou associação entre a realização de cirurgia de afirmação de gênero e o uso de álcool e/ou o tabagismo ao longo da vida. De todo modo, outros estudos demonstram que pessoas transgênero que não conseguem acessar a cirurgia de afirmação de gênero podem apresentar maior prevalência de consumo de drogas, em razão de fatores tanto psicológicos quanto sociais ^(^[xref ref-type="bibr" rid="r36"]^36^[/xref]^,^[xref ref-type="bibr" rid="r37"]^37^[/xref]^)^. A disforia de gênero não resolvida, associada ao sofrimento crônico pela incongruência entre corpo e identidade de gênero, contribui significativamente para sintomas de ansiedade, depressão e estresse, condições frequentemente relacionadas ao uso de substâncias lícitas e ilícitas ^(^[xref ref-type="bibr" rid="r37"]^37^[/xref]^,^[xref ref-type="bibr" rid="r38"]^38^[/xref]^)^. Além disso, barreiras estruturais dificultam o acesso à cirurgia de afirmação, ampliando a sensação de marginalização e de exclusão social por parte dessa população ^(^[xref ref-type="bibr" rid="r36"]^36^[/xref]^,^[xref ref-type="bibr" rid="r39"]^39^[/xref]^)^. Tais circunstâncias podem favorecer comportamentos de risco, incluindo o uso de drogas ilícitas, muitas vezes, como tentativa de alívio emocional temporário ^(^[xref ref-type="bibr" rid="r39"]^39^[/xref]^)^. Esses resultados reforçam as especificidades de saúde desse público e como elas podem impactar a sua saúde mental.

Este estudo apresenta algumas limitações. Primeiramente, por ser transversal, não permite estabelecer relações causais entre os eventos, embora tenha sido adotada uma análise criteriosa para reduzir vieses. A segunda limitação refere-se à predominância de homens transgênero na amostra, o que reflete sua maior demanda por atendimento em ambulatórios. Por fim, a terceira limitação está no uso de instrumentos validados para pessoas cisgênero. Tais instrumentos, baseados em concepções cisnormativas, não contemplam as vivências específicas de pessoas transgênero nem as complexidades sociais e culturais que impactam essa população. A escassez de instrumentos psicométricos adequados a esse grupo representa uma lacuna importante para a comunidade científica.

Apesar dessas limitações, destaca-se o uso do questionário ASSIST (teste de triagem para envolvimento com álcool, tabagismo e substâncias), validado e de fácil aplicação na atenção primária. Nesse sentido, os resultados indicam a necessidade de triagens rotineiras para transtornos de humor e uso de substâncias em populações vulneráveis. Além disso, evidenciam a urgência de estudos longitudinais que elucidem os mecanismos causais entre disforia de gênero, saúde mental e uso de drogas, bem como de ensaios clínicos que testam intervenções específicas para essa população. 

Em síntese, os resultados reforçam a alta vulnerabilidade de pessoas transgênero ao consumo de substâncias como tabaco, maconha e álcool. Variáveis socioeconômicas e de saúde associaram-se ao uso de álcool e de outras drogas, evidenciando a influência dos determinantes sociais. Também se destaca a urgência de eliminar barreiras no acesso à cirurgia de afirmação de gênero, medida que pode reduzir sofrimento psíquico e comportamentos de risco. Por fim, fica evidente que o consumo de substâncias nesse grupo decorre da interseção de fatores estruturais, emocionais e clínicos. Assim, os dados reforçam a importância de políticas públicas intersetoriais e de estratégias de cuidado integradas, inclusivas e sensíveis às desigualdades enfrentadas pela população transgênero.

## Data Availability

Os dados estão protegidos por questões éticas e poderão ser disponibilizados por solicitação ao autor correspondente.

## Material Suplementar

Tabela Suplementar
